# Raman and infrared spectroscopy reveal that proliferating and quiescent human fibroblast cells age by biochemically similar but not identical processes

**DOI:** 10.1371/journal.pone.0207380

**Published:** 2018-12-03

**Authors:** Katharina Eberhardt, Christian Matthäus, Shiva Marthandan, Stephan Diekmann, Jürgen Popp

**Affiliations:** 1 Spectroscopy and Imaging, Leibniz Institute of Photonic Technology, Jena, Germany; 2 Institute for Physical Chemistry and Abbe Center of Photonics, Friedrich Schiller University, Jena, Germany; 3 Department of Molecular Biology, Leibniz Institute on Aging – Fritz Lipmann Institute, Jena, Germany; Technische Universitat Munchen, TranslaTUM, GERMANY

## Abstract

Dermal fibroblast cells can adopt different cell states such as proliferation, quiescence, apoptosis or senescence, in order to ensure tissue homeostasis. Proliferating (dividing) cells pass through the phases of the cell cycle, while quiescent and senescent cells exist in a non-proliferating cell cycle-arrested state. However, the reversible quiescence state is in contrast to the irreversible senescence state. Long-term quiescent cells transit into senescence indicating that cells age also when not passing through the cell cycle. Here, by label-free *in vitro* vibrational spectroscopy, we studied the biomolecular composition of quiescent dermal fibroblast cells and compared them with those of proliferating and senescent cells. Spectra were examined by multivariate statistical analysis using a PLS-LDA classification model, revealing differences in the biomolecular composition between the cell states mainly associated with protein alterations (variations in the side chain residues of amino acids and protein secondary structure), but also within nucleic acids and lipids. We observed spectral changes in quiescent compared to proliferating cells, which increased with quiescence cultivation time. Raman and infrared spectroscopy, which yield complementary biochemical information, clearly distinguished contact-inhibited from serum-starved quiescent cells. Furthermore, the spectra displayed spectral differences between quiescent cells and proliferating cells, which had recovered from quiescence. This became more distinct with increasing quiescence cultivation time. When comparing proliferating, (in particular long-term) quiescent and senescent cells, we found that Raman as well as infrared spectroscopy can separate these three cellular states from each other due to differences in their biomolecular composition. Our spectroscopic analysis shows that proliferating and quiescent fibroblast cells age by similar but biochemically not identical processes. Despite their aging induced changes, over long time periods quiescent cells can return into the cell cycle. Finally however, the cell cycle arrest becomes irreversible indicating senescence.

## Introduction

Mostly, dermal fibroblasts are quiescent, a state of reversible cell cycle arrest [1]. During quiescence, cells are metabolically active, e.g. extracellular matrix proteins like collagen fibers are secreted [2]. Furthermore, tissue homeostasis and the maintenance of tissue function, among others, are controlled during quiescence, in order to prevent uncontrolled proliferation after returning into the cell cycle mode [3]. Quiescent cells are able to return into the cell cycle also after long periods of time. Wound-activated fibroblasts proliferate and migrate to wounds, coordinate the healing response by secretion of molecules and recruiting endothelial cells. Finally, they enter into quiescence again. Quiescence is associated with functional changes like modified metabolism [4] and alterations in chromatin conformation [5, 6]. Specific molecular mechanisms trigger quiescence actively [7], as for instance contact inhibition, presence or absence of nutrients, adhesion loss or mitogen withdrawal. All of these are associated with comprehensive changes in gene expressions or regulations of cell division genes [2]. Preventive protection from radical damages or transition into terminal differentiation constrain the reversible non-dividing state [8]. These processes result in slightly different quiescent states which however share a common genetic program [8–10]. To ensure the reversibility from quiescence into the cell cycling state, terminal differentiation (a further irreversible cell cycle arrest) has to be suppressed [8], which in turn enables tumor development. Cells protect themselves by short-term quiescence from a transition into senescence [10], whereas long-term quiescence induces a senescent-associated cell cycle arrest in order to prevent malignant transformations [9].

When aging, normal diploid fibroblasts irreversibly arrest the cell cycle and become senescent due to the loss of the proliferation potential [11]. Effects of cellular senescence are antagonistic due to tumor suppression in younger age and the significant involvement in the aging process in later life [12, 13]. Senescent cells display specific phenotypes, e.g. morphological changes like flattening and enlargement [14], different expression of specific molecular signatures and enhancement in senescence-associated β-galactosidase (SA β-Gal) activity [15]. These can be mediated by persistent DNA damage responses due to genotoxic stress (inducing premature senescence) or by modified telomere regulation due to unprotected telomeres (replicative senescence), also leading to persistent DNA damage responses [16]. If telomere shortening due to replication would be the dominant origin for replicative senescence and aging [17], long-term quiescence would be combined with an extended lifespan of fibroblast cells compared to continuous proliferation [18], since during long-term quiescence cells do not show telomere shortening. In contrast, during long-term quiescence, cells transit into senescence [9], contradicting the theory that telomere shortening is the main driving force for cellular aging [19]. Senescence and quiescence pathways share common features to ensure a cell cycle arrest [20, 21]. Here we asked to what extent aging, proliferating and long-term quiescent fibroblast cells show similar or distinct biochemical compositions. By label-free vibrational spectroscopy we studied quiescent, proliferating and replicatively senescent cells. Raman spectroscopy (RS) and infrared spectroscopy (IRS) have a high potential for classifying different cellular states (e.g. proliferation, terminal differentiation, apoptosis, aging or cancer) [22–27]. Raman and infrared spectra of individual cells are representative for their molecular compositions and can be seen as a unique molecular fingerprint. The spectra of quiescent cells were compared to those measured (i) in proliferating cells, (ii) after re-entry into proliferation after short- and long-term quiescence and (iii) senescent cells. We found that, during aging, proliferating and quiescent human fibroblast cells show biochemically similar but distinguishable changes.

## Materials and methods

### Cell cultivation

Primary human dermal fibroblasts from fetal foreskin (BJ; CRL-2522) were obtained from ATCC (Manassas, VA, USA) at young population doubling (PD) of 26–28. Cells were cultivated with 9.5% CO_2_ under humidified atmosphere at 37 °C in Dulbecco’s modified Eagles medium (DMEM) with L-Glutamine and low glucose (PAA Laboratories, Pasching, Austria). Medium was supplemented with 10% fetal bovine serum (FBS; PAA Laboratories, Pasching, Austria). Kanamycin (0.11 mM) and ampicillin (0.28 mM) were added into the incubation media to avoid contaminations during long-term experiments. All proliferating and quiescent cells were cultivated on autoclaved calcium fluoride (CaF_2_) slides (Crystal, Berlin, Germany), in order to avoid background scattering. Quiescent cells (PD 28) were respectively contact-inhibited (cultivation with normal media) or serum-starved (0.1% FBS in media) for a short- (0, 7 and 14 days) or long-term (100 days) cultivation. After 14 and 100 days of quiescent cultivation, cells recovered from quiescence were generated by splitting and cultivation under normal conditions for 3 additional days. Proliferating cells (PD 28) were cultivated for comparison. Senescence was induced by sub-cultivation: cells were washed with 1x phosphate-buffered saline (PBS, pH 7.4; PAA Laboratories, Pasching, Austria), detached by 0.05% Trypsin-EDTA (PAA Laboratories, Pasching, Austria) and splitted in a ratio of 1:4. Replicatively senescent cells (PD 70) were cultivated until the cell cycle arrest was microscopically observed by an increase in cell size, strongly reduced cell growth and confluency <50%. According to the various cell states, under each condition, 3 cell batches were generated. Following standard protocols, proliferating and quiescent cells were directly fixed with 4% paraformaldehyde in 1x PBS at room temperature for 10 minutes and senescent cells after 1 day adherence. Before and after fixation, cells were washed 3 times with 1x PBS. Fixed cells were stored at 6 °C for up to 2 weeks. Once cells were imaged by RS (3 batches), IR spectroscopic measurements (2 batches) and the SA β-Gal assay were performed (1 batch) as described by Dimri *et al*. [15].

### Raman spectroscopy

Raman images were recorded by a confocal Raman microscope (alpha300 R; WITec, Ulm, Germany) equipped with a 488 nm excitation laser, provided by a single mode diode laser. Laser light was focused onto the sample with ca. 10 mW intensity by using a 60x water dipping objective (NA 1.0; Nikon, Tokyo, Japan). The samples were scanned in the x-y plane in a raster pattern at a constant stage speed. Spectra were collected with 1 μm step size and an integration time of 1 second. The back-scattered Raman signal was recorded by a spectrometer, equipped with a 600 lines/mm grating and a -67 °C cooled EMCCD camera (Newton 970; Andor, Belfast, UK) with a 1600x200 array of 16 μm pixels. All 3 batches were measured and a minimal of 10 cells per batch was imaged.

### Infrared spectroscopy

Fourier-transform infrared spectroscopic (FT-IRS) measurements were performed (Varian 670-IR spectrometer; Agilent, Santa Clara, CA, USA) via transmission mode and 64 scans with a spectral resolution of 4 cm^-1^ were co-added. The spectra were acquired by a frequency range of 900–4000 cm^-1^ and carried out by a Mercury Cadmium Telluride detector. Background measurements were performed regularly to minimize noise and the water vapor signal. A home-built box around the microscope table enabled the samples to be purged by dry air before and during the measurements to reduce spectral contributions from water vapor. Cells from 2 of 3 batches were analyzed by using an aperture size of ca. 50 × 50 μm and a minimal of 100 cells per batch were recorded.

### Data analysis

The obtained data were pre-processed by using standard pre-processing algorithms accounting for noise reduction, baseline correction, cosmic ray removal (Raman spectroscopy) and atmospheric water subtraction (FT-IR spectroscopy). All pre-processing algorithms are available for the software “R” [28, 29]. “R” is an environment for statistical computing and graphics [30]. Mainly, the packages “hyperSpec” and “ggplot2” were used for data import, pre-processing, analysis and graphical visualization [28, 31].

#### Raman data

Images were cosmic spike removed [32]. Wavenumber deviations were corrected by aligning the Phenylalanine band to 1004 cm^-1^. All spectra underwent a smoothed interpolation and spectral truncation onto a new wavenumber axis from 400–3100 cm^-1^ with a data point spacing of 2 cm^-1^. Baseline correction was performed with an extended multiplicative signal correction (EMSC) to remove a linearity, an offset and water contributions. A mean spectrum was calculated from each cell which then was area normalized.

#### FT-IR data

Spectra were cut into the fingerprint (900–1800 cm^-1^) and high wavenumber region (CH-stretching; 2800–3100 cm^-1^). Subsequently, a linear baseline was fitted automatically to all spectra in the separated wavenumber regions. For noise reduction, the collapsed data were interpolated by smoothing with data points every 4 cm^-1^. Finally, the spectra were area normalized.

#### Statistics

To classify cells, a partial least squares linear discriminant analysis (PLS-LDA) was applied, implemented in the package “cbmodels”, which based on combinations of packages “pls” for PLS and “MASS” for LDA [33–35]. The data were described by PLS as a small number of latent variables that vary according to the property of interest and provide regularization for the subsequent LDA. Such a regression analysis proved to be suitable for statistical analysis of Raman and FT-IR spectra [36, 37]. For the Raman and FT-IR data, the number of latent variables was set to 4. In total, Raman spectra of 902 cells and FT-IR spectra of 2,351 cells were used for classification (S1 and S2 Tables). The PLS-LDA model was validated with a 10-fold cross validation with 100 iterations.

## Results

Proliferating fibroblasts transit into quiescence either by contact inhibition or serum starvation. Here, we mainly analyzed contact-inhibited quiescent cells, because same serum conditions allowed a direct comparison with proliferating and senescent cells. As an alternative to techniques based on molecular labeling (e.g. flow cytometry)–requiring extended sample preparations or destroying whole cells (e.g. Western blotting or RNA sequencing)–we applied label-free vibrational spectroscopy. As a reference, SA β-Gal activity of cells was measured.

### SA β-Gal staining

**Fig 1:**
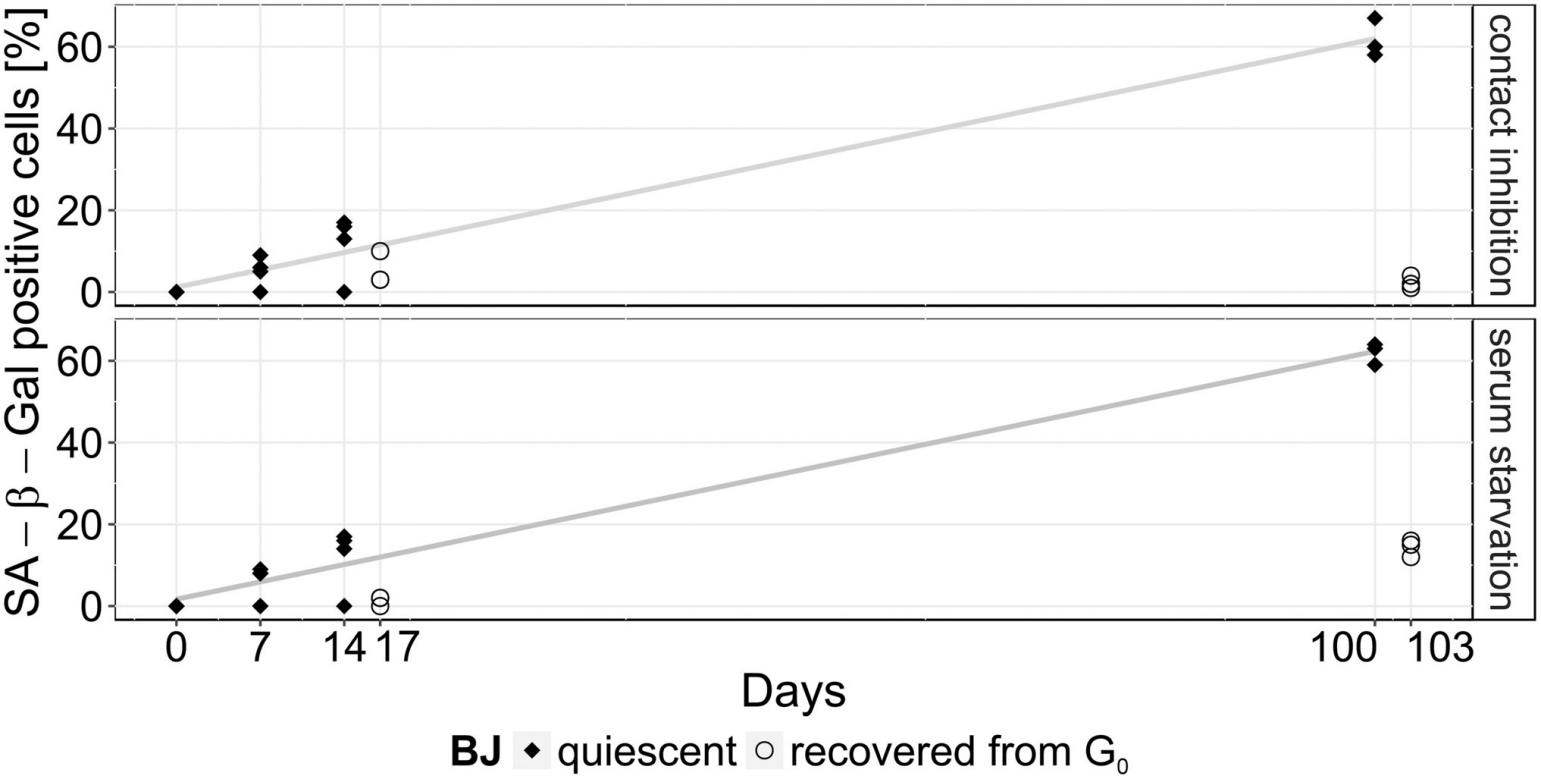
Number of positively SA β-Gal stained quiescent cells. Percentage of positively stained SA β-Gal fibroblasts during short- (0, 7 and 14 days) and long-term (100 days) cultivation of quiescent BJ cells (black dots) transferred by contact inhibition (top) or serum starvation (bottom). After 14 and 100 days, cells were cultivated for 3 days in total under normal proliferating conditions (“recovered from G_0_“; unfilled circles). Each data point represents a single experiment and the experimental error of all measured values is about ±10%.

Quiescence was induced in human fibroblast BJ cells either by contact inhibition or serum starvation. Subsequently, the percentage of SA β-Gal positive cells in culture were determined (Fig 1) [15, 38]. In BJ fibroblast cells, SA β-Gal staining is a good senescence marker [39, 40].

For short-term quiescence (0, 7 and 14 days), similar SA β-Gal values were observed, independent of both modes of quiescence induction: after 14 days up to 15% of the cells were tested positively for SA β-Gal. Cells recovered from quiescence, proliferated within the following 3 days and showed low levels of SA β-Gal staining: we detected <6% and <2% SA β-Gal positive cells in cell populations after quiescence induced by contact inhibition and serum starvation, respectively. Long-term quiescent cells (100 days) tested positively for ca. 60% SA β-Gal, which is consistent with our recent results for long-term quiescent MRC-5 and WI-38 cells [41]. This increase in SA β-Gal staining was independent of the mode of quiescence induction. Proliferating cells recovered from long-term quiescence, were tested positively for <5% and <15% SA β-Gal, when quiescence had been induced by contact inhibition or serum starvation, respectively. Constantly proliferating cells show no SA β-Gal activity upon staining in younger populations (PD 28; data not shown), but high SA β-Gal activity during senescence (> 85% SA β-Gal positive, maximum PDs of 70–72; data not shown) [39, 42]. SA β-Gal staining resulted in low numbers in proliferating cells which had recovered from quiescence. Recently, we observed that the proliferation marker Ki-67 strongly decreased with time down to about 30% for MRC-5 and WI-38 cells, being released from long-term quiescence, as well as for proliferating control cells [41]. Assuming a similar behavior for the BJ cells analyzed here, supports our interpretation that after 100 days SA β-Gal staining identified ca. 60% of the long-term quiescent as well as proliferating cells as being senescent, in other words being irreversibly cell cycle arrested. After release of quiescence induction of long-term quiescent cells, only the remaining cells (ca. 40% of these) are non-senescent and can start to proliferate again, resulting in the low SA β-Gal values measured after cell cycle revival, following long-term quiescence (Fig 1).

### Spectroscopic analysis and classification

Individual BJ fibroblasts were recorded in imaging mode. All Raman spectra of each single cell were averaged resulting in one mean spectrum of the cellular components for each cell. All spectral bands and features can be assigned to major cellular components (proteins, lipids and nucleic acids) [43, 44]. Most prominent Raman and FT-IR bands were assigned according to the S3 Table. Raman and FT-IR spectra were classified by PLS-LDA [36]. The algorithm was applied to identify spectral differences between: (i) the quiescence cultivation time (short- and long-term), (ii) two modes of quiescence induction (either contact inhibition or serum starvation), (iii) proliferating cells recovered from quiescence vs. quiescence and (iv) the cell states proliferation, senescence and quiescence amongst each other.

Quiescence was induced by contact inhibition or serum starvation. During the cultivation time, contact-inhibited short- and long-term cultivated cells were grown to full confluency without splitting the cell population. Serum-starved cells were characterized by less confluency, and, with ongoing cultivation, showed stronger morphological changes (in particular accumulations of lipid droplets (S1C Fig)) than contact-inhibited cells (especially observed by RS imaging; S1B Fig).

#### Cultivation time of quiescent cells

**Fig 2:**
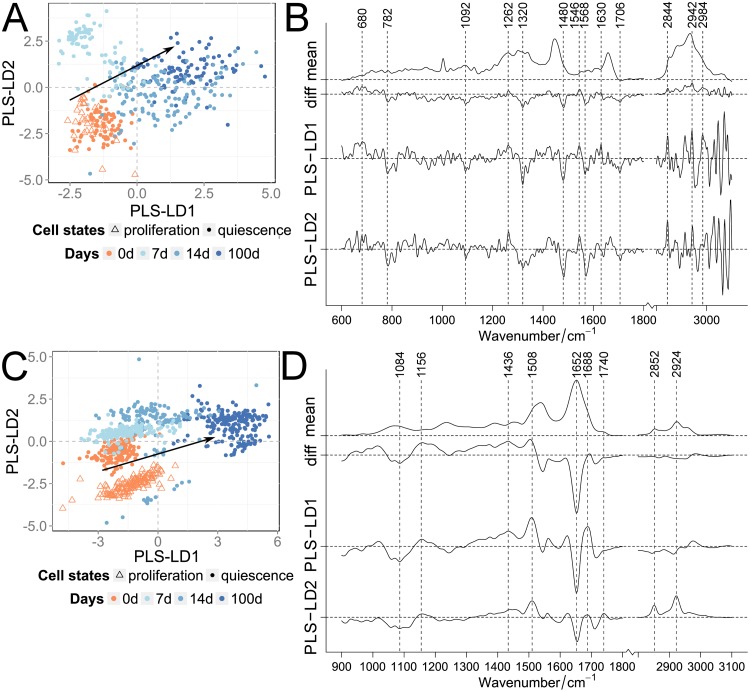
Classification of quiescent cells for the cultivation time. PLS-LDA of Raman (A, B) and FT-IR spectra (C, D) of proliferating and contact-inhibited quiescent cells separated by their cultivation time. Scatter plots (A, C) of proliferating (orange triangle) and quiescent cells after 0 days (orange dots) compared to 7, 14 and 100 days (blue dots; color darkens with increasing time). Arrows indicated the direction of the classified cultivation time. Mean and difference spectra (“diff”, 100 vs. 0 days) from the classified cells are plotted together with the PLS-LD1 and -LD2 coefficients vs. the wavenumber (B, D).

Contact-inhibited quiescent cells were long-term cultivated. RS and FT-IR spectra of shortly quiescence induced cells (0 days–beginning of quiescence) were compared with those obtained from quiescent cells after short-term (7 and 14 days) and long-term cultivation (100 days). The mean spectra of 0, 7, 14 and 100 days cultivated quiescent cells showed typical features of Raman and IR spectra of cells, which can all be assigned to the major cellular components such as proteins, lipids and nucleic acids (S2 Fig). These spectral characteristics exhibit considerable similarities, however minute but reproducible spectral differences can be discerned by common statistical algorithms. In order to analyze spectral differences induced by quiescence, the Raman and FT-IR data sets of quiescent cells were analyzed by a PLS-LDA algorithm previously described [36, 37]. In total 434 RS and 806 FT-IR cell spectra of the respective cultivation times were subjected to the analysis (Fig 2; S1 and S2 Tables).

The first four PLS components described the spectral variance of the Raman and FT-IR data with ca. 89 and 84%, respectively. Fig 2A and 2C show the score plots of the analysis for the Raman and FT-IR data. Both the RS as well as the FT-IR data, show a strong similarity between proliferating and quiescent cells right after quiescence induction (0 days; Fig 2A). With increasing cultivation times this was followed by a gradual increase in influence of both, PLS-LD1 and -LD2. LD1 very efficiently separated short- from long-term quiescent cells. For the FT-IR data, LD2 coefficients have a stronger influence on the discrimination of the short- vs. the long-term cultivated quiescent cells (0 days vs. 7, 14 and 100 days). The numbers of spectral outliers are high within the 7 and 14 days short-term cultivated cells compared to other cellular states. We believe that this fluctuation is due to the transition from proliferation to quiescence, which may vary amongst cells in duration of this transition and different expression levels of several genes [38]. The main contributions of the RS as well as FT-IR coefficients were associated with proteins and lipids. The LD1 and LD2 coefficients of the RS data analysis show intensity and band position variations within the amide I band, as well as in the lower CH-stretching region around 2850 cm^-1^. In addition, two very obvious intensity contributions at 1480 and 1568 cm^-1^ were observed. These bands cannot be interpreted as usual protein/lipid compositional differences, but likely arise from either increase or decrease of chromophores that are associated with the induced quiescence. The LD1 and LD2 coefficients of the FT-IR data set indicate very pronounced spectral differences in the amide I and II region, confirming the differences in the protein composition observed in the Raman spectra. Especially the differences in intensity and band position of the amide II region between 1500 and 1550 cm^-1^ was indicative for changes associated with structural proteins [45]. In both data sets also small differences were observed in the spectral region below 1100 cm^-1^. These might indicate differences in DNA/RNA compositions, as for instance the 782 cm^-1^ Raman band (usually observed at 785 cm^-1^) or the region between 1000 and 1100 cm^-1^, which is typical for deformations of the phosphate ester backbone, observed in RS as well as in FT-IR. A subsequent cross validation of contact-inhibited quiescent cells with ascending cultivation time provided a classification accuracy of >92%, and sensitivity and specificity were >85% (S5 Table). In the confusion table, more false predictions were determined for the transitional phases (7 and 14 days) of the FT-IR data (S4 Table), while for the Raman data non-consistent assignments were observed for 0 and 100 days.

In summary, RS and FT-IRS detected small differences in biomolecular composition in contact-inhibited quiescent cells, which gradually increased with cultivation time, consistent with the continuous increase in SA β-Gal activity (see Results SA β-Gal staining).

#### Comparison between contact inhibition and serum starvation as two modes of quiescence induction

**Fig 3:**
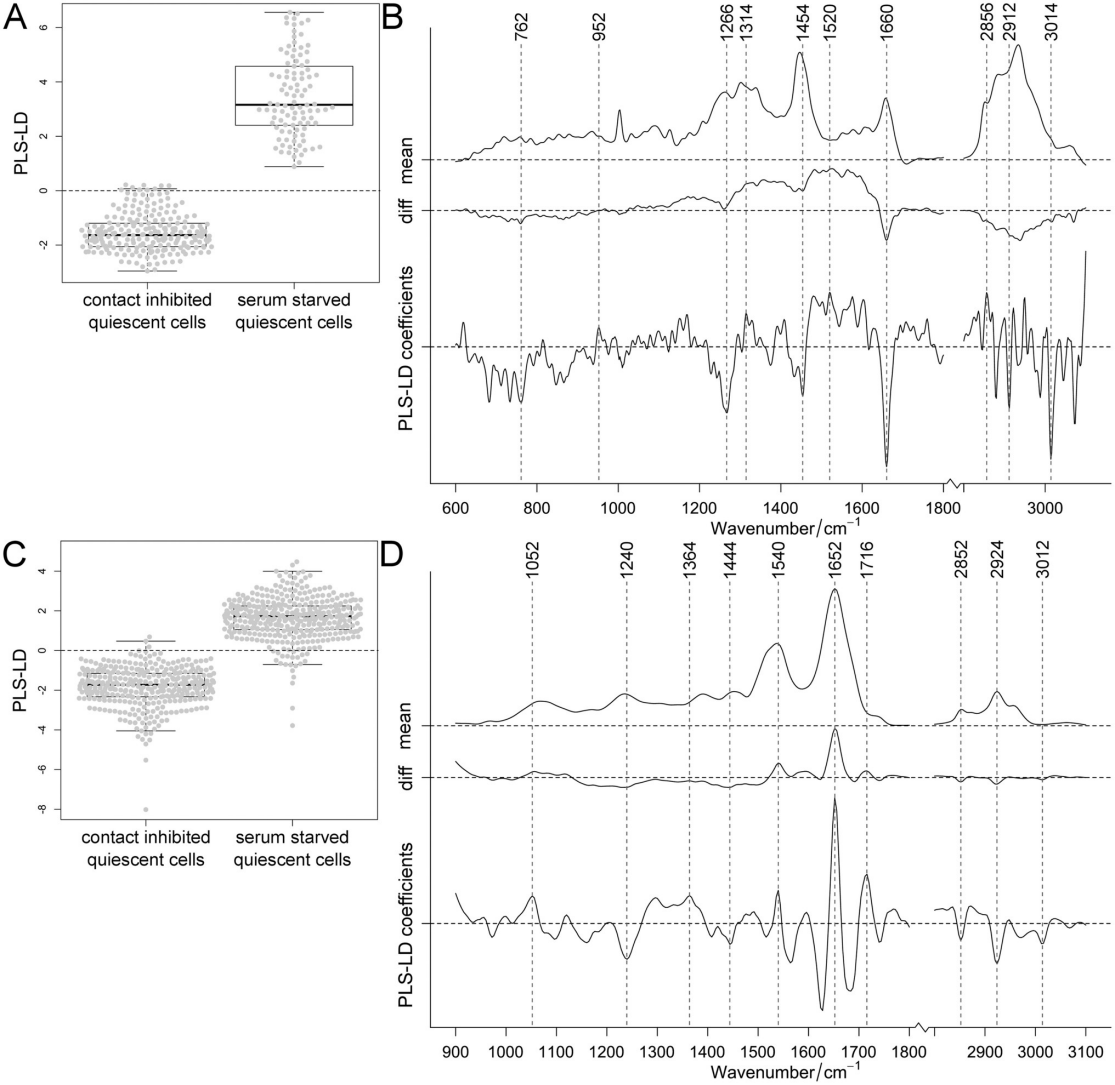
Classification of contact-inhibited and serum-starved quiescent cells. PLS-LDA of Raman (A, B) and FT-IR spectra (C, D) of cells in which quiescence was induced either by contact inhibition or by serum starvation (data sets for 14 and 100 days induction combined). Distribution of cells shown in one-dimensional scatter plots combined with box-plots (A, C) showing 95% confidence interval with p-values <0.001 for Raman and FT-IR data. Mean and difference spectra (“diff”, serum starvation vs. contact inhibition) from the classified cells are plotted together with the PLS-LD coefficients vs. the wavenumber (B, D).

Short- and long-term quiescence was induced by either (i) contact inhibition or (ii) serum starvation. Differences between the two modes of quiescence induction were observed in the collected Raman and FT-IR spectra, particularly within the fingerprint region (S3 Fig). With increased cultivation time, Raman intensities of the amide I (1640–1690 cm^-1^) and III band (1220–1300 cm^-1^) increased, while decreased intensities were observed in the high wavenumber region. In contrast, in the FT-IR fingerprint region, high spectral similarities were observed between the two modes of quiescence induction for both cultivation times (14 and 100 days). Classification of quiescent cells, either generated by contact inhibition or serum starvation, was performed for 332 Raman and 765 FT-IR spectra. The first four PLS components described the spectral variance of the Raman and FT-IR data with ca. 92 and 85%, respectively. The spectra of quiescent cells differed according to their mode of induction: positive coefficients of PLS-LDA were assigned to serum-starved and negative coefficients to contact-inhibited cells (separated by dashed line seen in Fig 3A and 3C).

Since only two groups were compared, which results in one linear discriminant, the outcome of the analysis was plotted in boxplots for a better visualization of the individual data points. The RS based classification was stronger than that by FT-IR (showing some overlap): the t-test analysis showed statistically significant differences. Since fewer cells were investigated by RS, serum-starved cells showed a larger variance in the Raman compared to the FT-IR data. In contact-inhibited quiescent cells, observed by RS, distinctive contributions of lipids (above 2800 cm^-1^) and proteins (1266, 1454 and 1660 cm^-1^) were identified. In comparison, serum-starved cells showed less prominent coefficients. In FT-IR, these cells displayed pronounced coefficients associated with proteins (1652 cm^-1^), while coefficients associated with contact-inhibited cells showed contributions from lipids (1240, 1740 and above 2800 cm^-1^) and proteins (1540 cm^-1^). Nonetheless, predominantly amide I bands were determined with both vibrational techniques. Cross validation of quiescent cells, induced either by contact inhibition or serum starvation, provided a classification accuracy of >96% and sensitivity and specificity were >95% (S6 Table). Taken together, vibrational spectroscopy distinguished modes of quiescence induction.

#### Proliferating cells recovered from quiescence

Next, we studied the proliferating capacity after short- and long-term quiescence (14 and 100 days, respectively). Contact-inhibited quiescent cells were passaged and isolated in order to induce proliferation. After 3 days of cultivation, a distinct proliferating capacity by the cell layer was observed, reaching a confluency of >80%. In these proliferating cells after being recovered from quiescence, cell size was similar to constantly proliferating control cells. Based on Raman images, lipid vesicle-like structures were observed within the cytosol, which were apparently associated with long-term incubations (S1B and S1C Fig). To some extent, cells, after being recovered from long-term quiescence by serum starvation, displayed morphological features similar to senescent cells, as for instance increased cell sizes, increased structures associated with lipids around the nuclei, and low confluency of less than <20% with a cellular growth arrest. This is consistent with a higher number of SA β-Gal staining of long-term quiescent cells recovered from serum starvation compared to those recovered from contact inhibition (Fig 1).

We analyzed in total 386 Raman and 694 FT-IR spectra of contact-inhibited quiescent cells and proliferating cells recovered from this state (S1 and S2 Tables). We detected slight differences (S4 Fig): after 14 days, the CH_2_-bending region (around 1446 cm^-1^) and the amide III bands (1302 and 1338 cm^-1^) showed slight differences in the Raman spectra. After 100 days cultivation, additional changes were observed in the amide I band (1658 cm^-1^) and around phenylalanine band at 1004 cm^-1^. The FT-IR spectra exhibit slight differences over the entire wavenumber region, but most prominently in the amide (1540 and 1652 cm^-1^) and high wavenumber region (2852 and 2924 cm^-1^). With increasing cultivation time spectral differences between contact-inhibited quiescent and from this quiescence recovered proliferating cells became more distinct.

**Fig 4:**
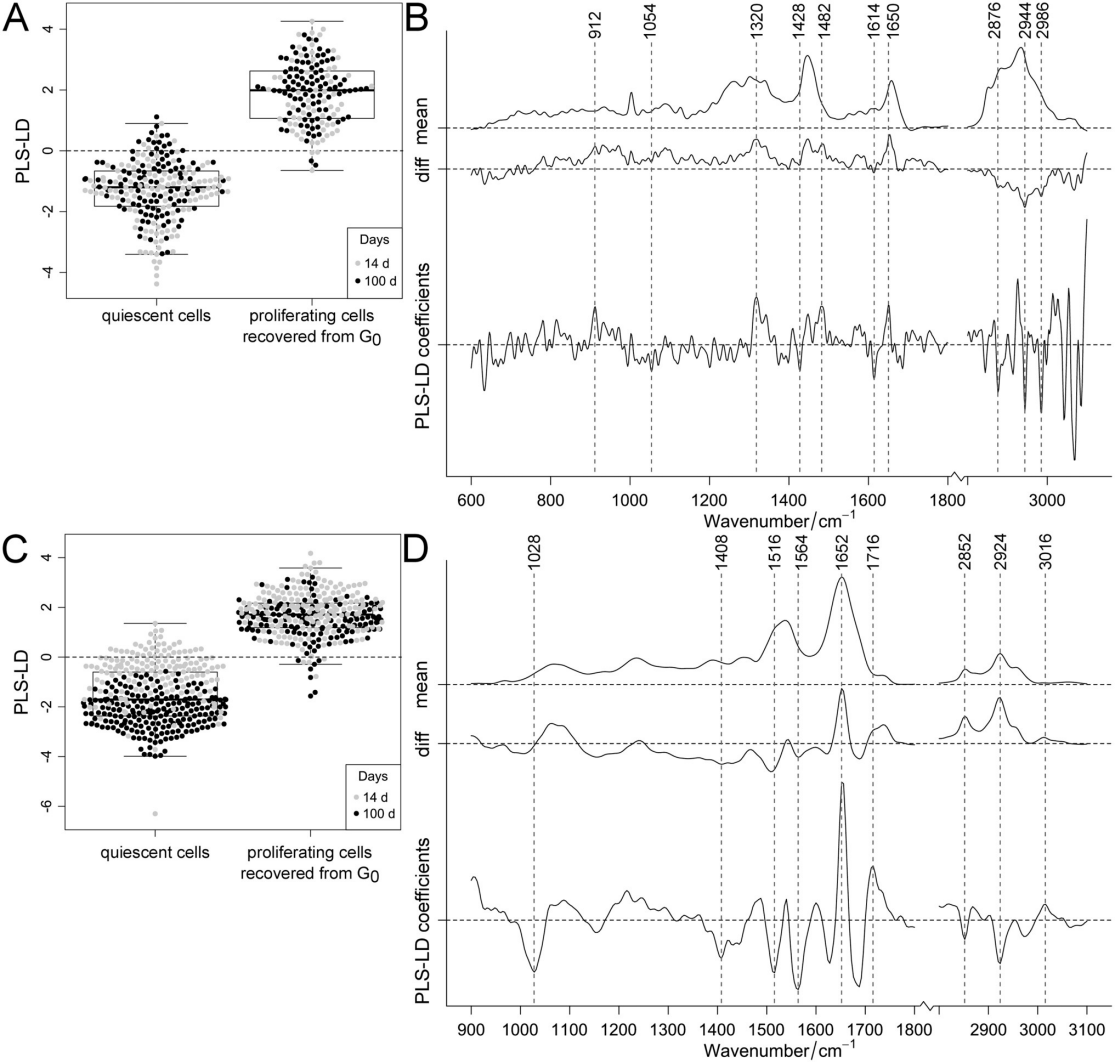
Classification of quiescent cells and proliferating cells recovered from quiescence. PLS-LDA of Raman (A, B) and FT-IR spectra (C, D) of contact-inhibited quiescent cells and proliferating cells recovered from quiescence after 14 and 100 days. Distribution of cells shown in one-dimensional scatter plots combined with box-plots (A, C) showing 95% confidence interval with p-values <0.001 for Raman and FT-IR data. Cultivation time was specified by color (14 days: gray, 100 days: black). Mean and difference spectra (“diff”, proliferating cells recovered from quiescence vs. quiescent cells) from the classified cells are plotted together with the PLS-LD coefficients vs. the wavenumber (B, D).

Four PLS components described a variance of ca. 85% and ca. 90% for the Raman and FT-IR data, respectively. The PLS-LDA was able to distinguish Raman and FT-IR spectra according to quiescence and proliferating cells, which were recovered from quiescence (Fig 4A and 4C).

Positive coefficients were assigned to (recovered) proliferating cells and negative coefficients to quiescent cells (separated by dashed lines seen in Fig 4A and 4C). The recovered cells showed nearly no separation according to the cultivation time. The associated PLS-LDA coefficients can therefore only be interpreted as a cell to cell variation that is not related to quiescence. Nevertheless, the data analysis of the FT-IR data of quiescent cells cultivated for 14 to 100 days, showed a clear trend for separation (Fig 4C, consistent with Fig 2C). The FT-IR coefficients associated with quiescence can be assigned to lipids, as for instance the intensities at 1428 cm^-1^ and the region above 2800 cm^-1^, as well as proteins around 1408, 1516, 1564, 1628 cm^-1^ (Fig 4B and 4D). Quiescent cells apparently exhibit altered protein structures as indicated by a shifted band position at 1688 cm^-1^ and carbohydrate accumulations (according to a dominant FT-IR band at 1028 cm^-1^). Generally, the overall appearance of the amide I and II profiles are completely altered. A cross validation of the FT-IR and RS data set revealed accuracy of >94% and the sensitivity and specificity of cells, recovered from quiescence, were >91% (S7 Table). Within the FT-IR data more superimpositions were noticed than in the Raman data. Overall, RS and FT-IRS distinguished quiescent cells from proliferating cells which have recovered from this state. With increasing cultivation time, spectral differences between these two cell groups became more distinct, mainly in compositional changes of lipids and proteins. Raman intensities and infrared absorptions were used to specify the differences in molecular abundance of quiescent cells compared to proliferating cells recovered form quiescence (S5 Fig). RS data at amide I (1658 cm^-1^) and III protein (1338 cm^-1^) bands displayed higher protein contents in recovered proliferating cells (S5A and S5B Fig). Also the FT-IR data showed an excess of amide I proteins (1652 cm^-1^) compared to protein and/or lipid contents with methyl or methylene groups (1446 cm^-1^) within the recovered cells (S5C Fig). Furthermore, within the recovered cells amide I was significantly higher compared to the amide II band (S5D Fig). Since no pure component was analyzed as control, the ratios only estimate the changes.

#### Cell state classification

In the context of cell differentiation based on spectral changes we compared Raman and FT-IR spectra of proliferating, quiescent and senescent cells. Raman imaging of individual fibroblasts also allows the observation of morphological characteristics. Quiescent cells grown to contact inhibition (>90% confluency) spread less than proliferating cells and were smaller in diameter. While proliferating cells have an average of ca. 80 μm, senescent cells grow to at least 150 μm. Furthermore, in senescent cells droplet-like accumulations of lipids around the nuclei were observed (S1D Fig). S6 Fig shows Raman and FT-IR mean spectra recorded for the different cell states. Over nearly the entire wavenumber region, Raman spectra of senescent cells clearly deviated from the other two rather similar spectra of proliferating and quiescent cells, while FT-IR spectra were quite similar to each other under these conditions (S6 Fig). Compared to earlier senescence measurements, differences within Raman spectra primarily depended on an altered and/or adapted baseline correction (data not shown). FT-IR spectra of proliferating and senescent cells measured here, were almost identical to previous results [36]. Thus, Raman spectra of senescent cells displayed clear differences not only to spectra of proliferating cells, confirming earlier studies, but also, as we show here, to long-term quiescent cells.

**Fig 5:**
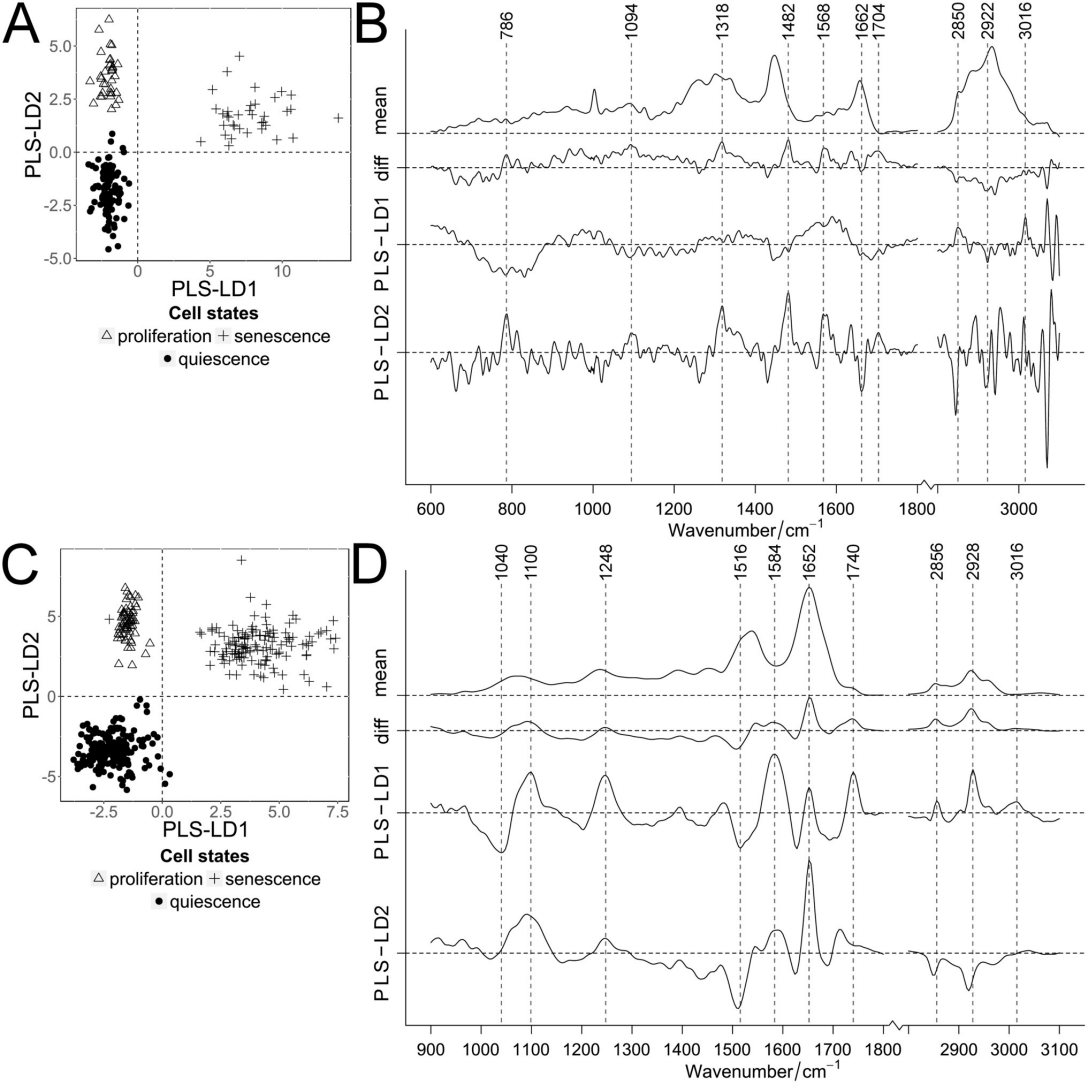
Classification of the 3 cell states: Proliferation, quiescence and senescence. PLS-LDA of Raman (A, B) and FT-IR spectra (C, D) of proliferating, quiescent and senescent cells. Scatter plots (A, C) of cell states: proliferation (triangle), senescence (plus) and quiescence (black dots, 100 days long-term cultivation by contact inhibition). Mean and difference spectra (“diff”, (B) proliferation vs. quiescence and (D) senescence vs. quiescence) from the classified cells are plotted together with the PLS-LD1 and -LD2 coefficients vs. the wavenumber (B, D).

Raman and FT-IR spectra of proliferating, senescent and long-term contact-inhibited quiescent cells were analyzed by regression analysis (in total 175 Raman and 479 FT-IR spectra; S1 and S2 Tables). The first four PLS components described the spectral variance of the Raman and FT-IR data with ca. 88% and 94%, respectively. Both data sets resulted in a distinct cell state separation (Fig 5A and 5C).

Although the spectral differences between proliferating and quiescent cells were smaller compared to their differences to senescent cells, the three cell states were clearly separated from each other by the PLS-LDA algorithm, independent of the mode of quiescent induction. Senescent and quiescence cells were differentiated from proliferating cells by PLS-LD1 and senescence was clearly distinguished from quiescence by LD2. Within the Raman data, LD1 was mostly background influenced while visible protein and lipid bands dominated LD2 (Fig 5B). On the other hand, in the FT-IR data LD1 displayed changes in amide II band indicative for structural proteins; whereas LD2 indicated changes in the amide I band (Fig 5D). Proliferating cells showed larger extents of α-helical structured proteins in FT-IR (1652 cm^-1^) compared to senescent cells, in which larger amounts of acidic amino acid associated bands (1584 cm^-1^) were observed. Obvious collagen-associated bands were not unambiguously identified. Also, in quiescent cells differences for proteins and lipids were detected. In FT-IR, LDA coefficients were associated with changes in the amide II band, predominantly around 1516 cm^-1^, and, to a lesser extent; with the amide I band (1624 cm^-1^). Lipid contributions were mainly observed in the high wavenumber regions (RS: 2850 and 2922 cm^-1^; FT-IR: 1436, 2856 and 2928 cm^-1^). Raman intensity ratios of protein bands (1652 cm^-1^, amide I) to lipids (1454 cm^-1^) and the differences within DNA and RNA (782 cm^-1^) were displayed for the different cell states (S7A and S7B Fig). Clear differences between contact inhibited quiescent and senescent cells were detected, the latter showing higher protein/lipid and lower nucleic acid contents. Proliferating cells had content values closer to quiescent cells. Senescent cells showed the lowest DNA/RNA content. Also FT-IR absorption bands were compared between amid I proteins (1652 cm^-1^) and proteins/lipids (1436 cm^-1^) as well as between lipids (1740 cm^-1^) and DNA/RNA (1716 cm^-1^) (S7C and S7D Fig). In quiescent compared to proliferating and senescent cells, protein contents (amide I) decreased and the amount of proteins with more methyl groups and/or lipids with more methylene groups increased. Compared to quiescent and proliferating cells, senescent cells contained the highest content of lipids and the lowest DNA/RNA content, while proliferating cells contained the highest amount of DNA/RNA. These results confirm the Raman data. Since no pure component (lipid, protein or DNA/RNA) was analyzed as control, the ratios are only an estimate of the molecular changes. Cross validation of proliferating, senescent and quiescent cells provided a classification accuracy of 99% of the PLS-LDA for each spectral data set (S8 Table). Sensitivity and specificity were >99%. Thus, RS as well as FT-IRS can separate these cellular states from each other; demonstrating that the biochemical composition in these cell states is sufficiently different from each other.

**Fig 6:**
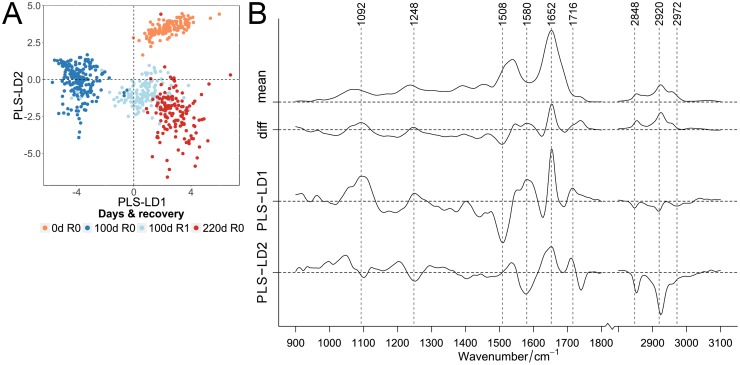
Classification of infrared spectra of proliferating, long-term cultivated quiescent and senescent cells. PLS-LDA scatter plot of FT-IR spectra (A) of proliferating cells (“0d R0”) and 100 days contact-inhibited quiescent cells without recovery (“100d R0”) and recovered from quiescence (“100d R1”) compared to replicative-grown senescent cells (“220 d R0”). Cultivation time (0, 100 and 220 days) and proliferating cells recovered from quiescence (“R1”, pale blue) were specified by color (0 days: orange, 100 days: blue, 220 days: red). Mean and difference spectra (“diff”, 220 d senescence vs. 100 d quiescence “R0”) from the classified cells are plotted together with the PLS-LD1 and -LD2 coefficients vs. the wavenumber (B).

If asked whether it is possible to directly find spectral indications that long-term quiescent cells show molecular signs of senescence, it is suitable to compare FT-IR spectra of proliferating cells (0 d) with (i) long-term (100 d) quiescent cells, with (ii) proliferating cells recovered from long-term (100 d) contact inhibition induced quiescence, and (iii) fully replicative senescent (220 d) cells. The proliferating cells, the long-term quiescent cells and the proliferating cells recovered from long-term quiescence can be clearly separated by the PLS-LDA algorithm (Fig 6A).

In contrast, the senescent cells displayed considerable overlap with proliferating cells recovered from quiescence, but neither with young proliferating nor with quiescent cells. These FT-IR data indicate that, indeed while being quiescent, cells develop molecular signatures of senescence. These indications are less clear in the corresponding Raman data (S8 Fig). Furthermore, our data showed that quiescence is not a state in which the cells remain biochemically invariant; when they return back into the cell cycle they are biochemically modified, showing an ageing associated imprint. Instead, during (long-term) quiescence the cells change their biochemical status, meaning during quiescence cells age and adopt biomolecular imprints similar to senescence [9]. In FT-IR coefficients, prominent protein bands can be assigned to amino acid side-chain vibrations (1508 and 1580 cm^-1^) and amide I with α-helical structures (1652 cm^-1^) (Fig 6B). Further assignments are possible for fatty acid esters (ester functional groups at 1740 cm^-1^ and symmetric and anti-symmetric CH-stretching at 2848 and 2920 cm^-1^) and nucleic acids (1092 and 1716 cm^-1^). Interestingly, PLS-LD1 was similar to difference spectra of senescence compared with quiescent cells, whereas LD2 agrees more with the recovered and non-recovered cells, suggesting that proteins, more than other biomolecules, influence this differentiation.

## Discussion

Cellular quiescence has been studied in detail by various biomolecular methods such as flow cytometry to analyze for cell cycle distributions, immunoblotting for monitoring of specific proteins or microarray analysis to compare gene expression profiles in proliferating and quiescent cells [4, 8–10, 46]. One of the commonly applied methodologies is deep RNA sequencing of proliferating, quiescent [41] and senescent cells [39, 42], which indicated changes in the mRNA expression profiles. On the genetic level, age-associated changes of various fibroblast cell strains exhibit strong conservations in the transcriptome. To identify unknown molecular structures, regarding the lipidome and metabolome on the single cell level, label-free vibrational RS and FT-IRS was used to study and compare the biomolecular composition of three cellular states of human fibroblast cells: proliferation, quiescence and senescence. Differences among these states due to composition and modification of biomolecules were detected. We observed differences in the associated spectral information between these cellular states over a large wavenumber region. Moreover, no single and dominant marker band was detected. The separation of proliferating and senescent cells observed here, is consistent with our earlier data [36, 37]. Especially in the FT-IR data, protein bands (amide I and II between 1500–1700 cm^-1^) decreased and a lipid band (1740 cm^-1^) increased with senescence, which is in the agreement with previous experiments noticeably matching the PLS-LDA results [36]. Vibrational spectroscopy clearly distinguished contact-inhibited from serum-starved quiescent cells. With increasing quiescence cultivation time, in the Raman and FT-IR spectra we observed continuous spectral shifts in the biomolecular composition in contact-inhibited quiescent cells, consistent with the increase in SA β-Gal activity. The spectra displayed spectral differences between quiescent and proliferating cells, recovered from quiescence, which became more distinct with increasing quiescence cultivation time. When comparing proliferating, quiescent and senescent cells, we found that RS as well as FT-IRS can differentiate between these cellular states. In long-term cultivated quiescent cells, we found indications of increased amounts of aromatic amino acids and less β-sheet structures in proteins, while in contrast, in proliferating cells more α-helical structures with less aromatic amino acids were detected. Basic amino acids like lysine exist in α-helikal structures in the alkaline pH range, whereas β-sheet structures were found in acidic environments [47, 48]. Denaturation of native helical proteins lead to aggregation of proteins with non-native β-sheets, which were clearly distinguishable in FT-IR. In senescent cells we observed increased acidic amino acids and less α-helical structures. During replicative senescence, lysosomal expressed enzymes (like SA β-Gal) are active within acidic pH values [15]. Also enzymes for protein degradation in lysosomes act in an acidic environment. Hence, within the analyzed cell states and transitions between them, protein conformational changes and modifications due to the cellular environment could be well detected, and allowed the separation amongst the cellular states. Furthermore, during transition into senescence, protein synthesis and proteolysis is diminished, proteasome activity is reduced [49, 50], and products from protein oxidation accumulate [49, 51]. Degradation of protein aggregates is disturbed due to oxidized and cross-linked proteins, e.g. fluorescent lipofuscin. Such fluorescence, as signs of damage, were especially observed in Raman spectra of senescent cells [36], but here also in long-term cultivated quiescent cells.

The quiescent state is based on the expression of a number of genes involved in the arrest of growth and division, regardless of the induction signal [8]. Here, we detected differences in the Raman and FT-IR spectra of quiescent cells after contact inhibition and serum starvation. Other than contact inhibition, serum starvation changes the nutrient conditions of cells. This results in a considerable reprogramming [52], which influences the biomolecular abundance [52, 53] and explains the spectral differences between the two modes of quiescence induction. Short-term quiescent cells are protected from becoming senescent by expression of HES1 [10]. However, with time, biomolecular damages occur, which in turn induce aging in the majority of cells in the absence of repair mechanisms [40, 41]. DNA damage accumulates not only in aging proliferating cells (transiting into senescence), but also during long-term quiescence at physiological oxygen levels [41]. DNA repair is more successful in proliferating cells recovered from short-term than from long-term quiescence. Despite their different modes of activation, some senescent and quiescent signaling pathways were found to be similar, such as downregulation of DNA repair genes [39, 42, 54]. Our Raman and FT-IR spectroscopic data support and extend this observation: with increasing cultivation time of quiescent cells, successive spectral shifts indicated continuous compositional changes during quiescence, similar but not identical to aging proliferating cells [39, 42]. This constant drift in biochemical compositions is supported by the observation that the longer the cell resumed in quiescence, the longer it takes for the cell to return to proliferation after release from quiescence [55–57], correlating with cell size [58]. After long-term quiescence and recovery, the proliferating cells transited into full senescence after only a few population doublings [41].

The transition from proliferation to senescence has been associated with telomere shortening due to replication in each cell division. Since (long-term cultivated) quiescent cells do not replicate and do not show telomere shortening [59–61], but nevertheless gradually become senescent [9], other, telomere length independent [62], potentially maintenance-driven mechanisms, like genotoxic stress, must induce cell cycle arrest [60, 61]. Reactive oxygen species are expected to contribute to these processes [63, 64]. Quiescence related cell cycle arrest was presumed to be independent of the senescence associated p53 signaling pathway [65]. This could explain, among others, the clear differentiation between senescent and quiescent cells observed here.

During quiescence, intracellular biomolecules can be degraded by an auto-phagocytotic process which prevents accumulation of damaged molecules [4, 66]. With increased activity of a proliferation-regulated protein (mechanistic Target of Rapamycin, mTOR), less auto-phagocytosis was observed in tissue containing senescent cells, despite increased lysosomal levels and lipofuscin [67]. In addition to autophagy, mTOR controls various other cellular activities including cell growth, mitochondrial metabolism or maintenance of the lysosomal function [68]. The lysosomal cellular content increases during senescence and quiescence due to lysosomal biogenesis, and enhances the SA β-Gal expression [67]. This process requires high mTOR activities. Thus, SA β-Gal intensities are lower in quiescent cells compared to senescent cells [41], as observed here. Generally, mTOR seems to assume a key role in the transition into quiescence and senescence. Inhibition of mTOR (by p53) favors quiescence, which prevents typical senescent phenotypes such as hypertrophy of the cytoplasm [69].

We have demonstrated the ability of RS and FT-IRS to differentiate various human cell states and the type of generation at the single cell level, in a label-free manner. Despite only minute biochemical differences between proliferating, senescent and quiescent cells, sophisticated statistical algorithms such as PLS-LDA are able to classify these cell states into separate groups. These vibrational techniques offer a promising way to identify different cell states–also cell types–within tissue *in vivo*. This could contribute to skin studies in future, especially with respect to the distinction of early tumorigenesis.

## Supporting information

S1 Table Number of cells imaged with Raman spectroscopy. Amount of analyzed fibroblast cells for PLS-LDA classification after RS imaging. ^a^ Proliferating cells recovered (“R”) from quiescence.
(DOCX)

S2 Table Number of cells analyzed with infrared spectroscopy. Amount of analyzed fibroblast cells for PLS-LDA classification after FT-IRS. ^a^ Proliferating cells recovered (“R”) from quiescence.
(DOCX)

S3 Table Band assignments of Raman (left) and FT-IR spectra (right), as described in the literature [43, 44]. ν—stretching; δ—deformation; δ_s_—scissoring; γ_t_—twisting; γ_w_—wagging; ρ—bending; sym.—symmetric; asym.—asymmetric.
(DOCX)

S4 Table Cross-validation of Raman and infrared spectra for the cultivation times. Ten-fold cross-validation of PCA-LDA with 100 iterations for the cultivation time (0, 7, 14 and 100 days) of contact inhibited quiescent cells without proliferating cells recovered from quiescence. Values for the Raman (“RS”) and FT-IR data are given in percentage.
(DOCX)

S5 Table Confusion table of Raman and infrared spectra for the cultivation times. Confusion table of Raman (“RS”) and FT-IR data for the PCA-LDA classification model for the cultivation times (in days) of contact inhibited quiescent cells.
(DOCX)

S6 Table Cross-validation of Raman and infrared spectra for the type of quiescent induction. Ten-fold cross-validation of PLS-LDA with 100 iterations for the kind of quiescent induction (contact inhibition or serum starvation) after 14 and 100 days without proliferating cells recovered from quiescence. Values for the Raman (“RS”) and FT-IR data are given in percentage.
(DOCX)

S7 Table Cross-validation of Raman and infrared spectra of proliferating cells recovered from quiescence. Ten-fold cross-validation of PLS-LDA with 100 iterations of contact inhibited quiescent cells and the same cells recovered from G_0_ phase after 14 and 100 days. Values for the Raman (“RS”) and FT-IR data are given in percentage.
(DOCX)

S8 Table Cross-validation of Raman and infrared spectra of three cell states. Ten-fold cross-validation of PLS-LDA with 100 iterations for the cell states (proliferation, senescence and 100 days contact inhibited quiescent cells) without proliferating cells recovered from quiescence. Values for the Raman (“RS”) and FT-IR data are given in percentage.
(DOCX)

S1 Fig Raman images of three fibroblast cell states. BJ cell states: (A) a proliferating cell (PD 28), (B) contact inhibited quiescent cells (100 days cultivation), (C) a serum starved quiescent cell (100 days cultivation) and (D) a senescent cell (PD 70). Images based on the C-H stretching region (2800 to 3020 cm^-1^) and the scale bars are (A) 5 μm and (B–D) 10 μm.
(DOCX)

S2 Fig Raman and infrared spectra of quiescent cells with various cultivation times. Mean and standard deviation of (A) Raman and (B) FT-IR spectra of contact inhibited quiescent cells (BJ PD 28) for the cultivation times 0, 7, 14 and 100 days. The 0, 7, 14 and 100 days cultivated cells were displayed by different line styles. For a better visualization, the low wavenumber region from 600–1800 cm^-1^ in (A) is plotted 3fold enhanced.
(DOCX)

S3 Fig Raman and infrared spectra for the type of quiescent induction. Mean and standard deviation of (A) Raman and (B) FT-IR spectra of contact inhibited (dotted line) and serum starved (solid line) quiescent fibroblast cells (BJ PD 28) after 14 days (top) and 100 days (below) cultivation. For a better visualization, the low wavenumber region from 600–1800 cm^-1^ in (A) is plotted enhanced 3fold.
(DOCX)

S4 Fig Raman and infrared spectra of proliferating cells recovered from quiescence versus quiescence. Mean and standard deviation of (A) Raman and (B) FT-IR spectra of contact inhibited quiescent cells (dotted line) and the same cells after recovery from quiescence (solid line) after 14 days (top) and 100 days (bottom) cultivation. The standard deviation is in gray (darker for quiescent cells and brighter for once again proliferating cells) and less pronounced. For a better visualization the low wavenumber region from 600–1800 cm^-1^ in (A) is plotted 3fold enhanced.
(DOCX)

S5 Fig Raman and infrared spectroscopy ratio analyses of mostly proteins for quiescent cells and proliferating cells recovered from quiescence. For quiescent cells (14 and 100 days contact inhibition) and proliferating cells recovered from quiescence (after 14 and 100 days contact inhibition, cells proliferating for 3 days), the 1658 cm^−1^ Raman band intensities (A, amide I proteins, C = C stretch) were plotted (with a fitted linear calibration, R^2^ = 0.17). Also, the 1338 cm^-1^ Raman band intensities (B, amide III proteins) were plotted (with a fitted linear calibration, R^2^ = 0.25). In total, 386 spectra were used for (A) and (B). Furthermore, in (C) FT-IR the absorption band at 1652 cm^-1^ (amide I, proteins) was related to 1446 cm^-1^ (proteins (asymmetric bending of methyl groups (CH_3_)) and/or lipids (CH_2_ scissoring of acyl chains)). In (D), FT-IR band ratios of 1652 cm^-1^ (amide I, proteins) versus 1540 cm^-1^ (amide II) are displayed. A linear calibration was fitted for (C, R^2^ = 0.41) and for (D, R^2^ = 0.53). In total, 694 spectra were used for (C) and (D).
(DOCX)

S6 Fig Raman and infrared spectra of three cell states. Mean and standard deviation of (A) Raman and (B) FT-IR spectra of proliferating (BJ PD 28; dotted line), senescent (BJ PD 70; dashed line) and quiescent fibroblast cells (BJ PD 28 after 100 days contact inhibition; solid line). For a better visualization the low wavenumber region from 600–1800 cm^-1^ in (A) is plotted enhanced three-fold.
(DOCX)

S7 Fig Raman and infrared spectroscopy ratio analyses for proteins, lipids and nucleic acids for the three cell states quiescence (100 days contact inhibited quiescent cells without recovery), proliferation and senescence. The Raman intensity ratio (A) of the 1652 cm^−1^ band (amide I proteins, C = C stretch) to the 1454 cm^−1^ band (lipids, CH_2_ twist) is plotted with a fitted linear calibration (R^2^ = 0.33). Also for the DNA/RNA intensity (B) the comparison of the three cell states is plotted using the Raman band 782 cm^-1^ (cytosine, thymine & uracil ring breathing) with a fitted linear calibration (R^2^ = 0.66). In total, 253 spectra were used for (A) and (B). Furthermore, in (C) FT-IR the absorption band at 1652 cm^-1^ (amide I, proteins) was related to 1436 cm^-1^ (proteins (asymmetric bending of methyl groups (CH_3_)) and/or lipids (CH_2_ scissoring of acyl chains)). In (D), FT-IR band ratios of 1740 cm^-1^ (C = O stretching of ester functional groups, lipids) versus 1716 cm^-1^ (C = O stretching of base pairing in nucleic acids, RNA/DNA contents) were displayed. A linear calibration is fitted for (C, R^2^ = 0.27) and (D, R^2^ = 0.65). In total, 627 spectra were used for (C) and (D).
(DOCX)

S8 Fig Classification of Raman spectra of proliferating, long-term cultivated quiescent and senescent cells. PLS-LDA scatter plot of Raman spectra (A) of proliferating cells (“0 d R0”) and 100 days contact-inhibited quiescent cells without recovery (“100 d R0”) and recovered from quiescence (“100 d R1”) compared to replicative-grown senescent cells (“220 d R0”). Cultivation time (0, 100 and 220 days) and proliferating cells having recovered from quiescence (“R1”, pale blue) were specified by color (0 days: orange, 100 days: blue, 220 days: red). Mean and difference spectra (“diff”, 0 d proliferation vs. 100 d quiescence “R1”) from the classified cells are plotted together with the PLS-LD1 and -LD2 coefficients vs. the wavenumber (B).
(DOCX)
