# Supplementary material for: Raman and infrared spectroscopy reveal that proliferating and quiescent human fibroblast cells age by biochemically similar but not identical processes
Source: PLoS One. 2018 Dec 3;13(12):e0207380. doi: 10.1371/journal.pone.0207380 (PMC6277109; doi:10.1371/journal.pone.0207380)
Supplement: S1 Table — Amount of analyzed fibroblast cells for PLS-LDA classification after RS imaging. a Proliferating cells recovered (“R”) from quiescence. (DOCX) [file pone.0207380.s001.docx]

**S1 Table. Number of cells imaged with Raman spectroscopy.**

| cell states | | days | | | | | | |  |
| --- | --- | --- | --- | --- | --- | --- | --- | --- | --- |
|  |  | 0 | 7 | 14 | 14 R^a^ | 100 | 100 R^a^ | 220 | in total |
| quiescence | contact inhibition | 78 | 92 | 125 | 61 | 105 | 95 |  | 556 |
|  | serum starvation | 47 | 55 | 53 | 35 | 49 | 37 |  | 276 |
| proliferation | | 34 |  |  |  |  |  |  | 34 |
| senescence | |  |  |  |  |  |  | 36 | 36 |
| in total | | 159 | 147 | 178 | 96 | 154 | 132 | 36 | 902 |

Amount of analyzed fibroblast cells for PLS-LDA classification after RS imaging.

^a^ Proliferating cells recovered (“R”) from quiescence.
