# Supplementary material for: Raman and infrared spectroscopy reveal that proliferating and quiescent human fibroblast cells age by biochemically similar but not identical processes
Source: PLoS One. 2018 Dec 3;13(12):e0207380. doi: 10.1371/journal.pone.0207380 (PMC6277109; doi:10.1371/journal.pone.0207380)
Supplement: S2 Table — Amount of analyzed fibroblast cells for PLS-LDA classification after FT-IRS. a Proliferating cells recovered (“R”) from quiescence. (DOCX) [file pone.0207380.s002.docx]

**S2 Table. Number of cells analyzed with infrared spectroscopy.**

| cell states | | days | | | | | | |  |
| --- | --- | --- | --- | --- | --- | --- | --- | --- | --- |
|  |  | 0 | 7 | 14 | 14 R^a^ | 100 | 100 R^a^ | 220 | in total |
| quiescence | contact inhibition | 148 | 164 | 151 | 217 | 211 | 146 |  | 1,037 |
|  | serum starvation | 159 | 112 | 124 | 239 | 279 | 133 |  | 1,046 |
| proliferation |  | 132 |  |  |  |  |  |  | 132 |
| senescence |  |  |  |  |  |  |  | 136 | 136 |
| in total |  | 439 | 276 | 275 | 456 | 490 | 279 | 136 | 2,351 |

Amount of analyzed fibroblast cells for PLS-LDA classification after FT-IRS.

^a^ Proliferating cells recovered (“R”) from quiescence.
