# Supplementary material for: Raman and infrared spectroscopy reveal that proliferating and quiescent human fibroblast cells age by biochemically similar but not identical processes
Source: PLoS One. 2018 Dec 3;13(12):e0207380. doi: 10.1371/journal.pone.0207380 (PMC6277109; doi:10.1371/journal.pone.0207380)
Supplement: S3 Table — ν—stretching; δ—deformation; δs—scissoring; γt—twisting; γw—wagging; ρ—bending; sym.—symmetric; asym.—asymmetric. (DOCX) [file pone.0207380.s003.docx]

**S3 Table. Band assignments of Raman (left) and FT-IR spectra (right), as described in the literature [41, 42].**

| Raman wavenumber [cm^‑1^] | Band assignments | Biomolecular classifications | FT-IR wavenumber [cm^‑1^] | Band assignments | Biomolecular classifications |
| --- | --- | --- | --- | --- | --- |
| 2800 – 3020 | ν(CH, CH_2_ & CH_3_) sym. & asym. | lipids, proteins | **2800 – 3100** | ν(CH, CH_2_ & CH_3_)  sym. & asym. | lipids, proteins |
| 1640 – 1690 | amide I (α‑helix), ν(C=C & C=O) | proteins | **1740** | ν(C=O) | lipids |
| 1564 – 1568 | ν(CN), ν(C=C), δ(NH), COO^-^ | proteins | **1716** | ν(C=O) | nucleic acids |
| 1480 | ν(CN), δ(NH), guanine & adenine ring breathing | lipids, proteins, nucleic acids | **1600 – 1700** | amide I (α-helix or β‑structure) | proteins |
| 1446 – 1454 | ν(CH), δ (CH_2_ & CH_3_) | proteins, lipids | **1580 – 1584** | ν(COO^-^) | amino acids |
| 1220 – 1340 | amide III, δ(CH_2_), ν(CN) & δ(NH),  ring breathing | proteins, collagen, lipids, DNA, RNA | **1500 – 1550** | amide II (α‑helix or β‑structure) | proteins |
| 1000 – 1100 | ν(PO_2_^‑^),  ν(C‑C & C‑O),  δ(OCH) | DNA, RNA, lipids, proteins | **1436 – 1446** | ρ(CH_3_) asym., δ_s_(CH_2_) | proteins, lipids |
| 1004 | ring breathing | protein (phenylalanine) | **~1408** | ρ(CH_2_ & CH_3_) | proteins |
| 782 – 785 | pyrimidine & uracil ring breathing | DNA, RNA | **1240** | ν(P=O) asym. | lipids, proteins |
|  |  |  | **1000 – 1100** | δ phosphate ester backbone, ν(C-O) | DNA, RNA, nucleic acids |
|  |  |  | **900 – 1200** | ν(P=O) sym.,  C-O-C & C-O ring vibrations,  C-O-P, P-O-P | nucleic acid; carbohydrates |

ν - stretching; δ - deformation; δ_s_ - scissoring; γ_t_ - twisting; γ_w_ - wagging; ρ – bending;
sym. - symmetric; asym. - asymmetric.
