# Supplementary material for: Raman and infrared spectroscopy reveal that proliferating and quiescent human fibroblast cells age by biochemically similar but not identical processes
Source: PLoS One. 2018 Dec 3;13(12):e0207380. doi: 10.1371/journal.pone.0207380 (PMC6277109; doi:10.1371/journal.pone.0207380)
Supplement: S4 Table — Ten-fold cross-validation of PCA-LDA with 100 iterations for the cultivation time (0, 7, 14 and 100 days) of contact inhibited quiescent cells without proliferating cells recovered from quiescence. Values for the Raman (“RS”) and FT-IR data are given in percentage. (DOCX) [file pone.0207380.s004.docx]

**S4 Table. Cross-validation of Raman and infrared spectra for the cultivation times.**

|  | accuracy |  | 0 days | 7 days | 14 days | 100 days |
| --- | --- | --- | --- | --- | --- | --- |
| RS | 98.3 | sensitivity | 100.0 | 95.6 | 95.0 | 99.0 |
|  |  | specificity | 99.0 | 99.7 | 100.0 | 97.5 |
| FT-IR | 92.1 | sensitivity | 88.6 | 85.0 | 88.0 | 97.6 |
|  |  | specificity | 99.4 | 94.8 | 94.1 | 99.5 |

Ten-fold cross-validation of PCA-LDA with 100 iterations for the cultivation time (0, 7, 14 and 100 days) of contact inhibited quiescent cells without proliferating cells recovered from quiescence. Values for the Raman (“RS”) and FT-IR data are given in percentage.
