# Supplementary material for: Raman and infrared spectroscopy reveal that proliferating and quiescent human fibroblast cells age by biochemically similar but not identical processes
Source: PLoS One. 2018 Dec 3;13(12):e0207380. doi: 10.1371/journal.pone.0207380 (PMC6277109; doi:10.1371/journal.pone.0207380)
Supplement: S5 Table — Confusion table of Raman (“RS”) and FT-IR data for the PCA-LDA classification model for the cultivation times (in days) of contact inhibited quiescent cells. (DOCX) [file pone.0207380.s005.docx]

**S5 Table. Confusion table of Raman and infrared spectra for the cultivation times.**

| RS | | | | | FT-IR | | | | |
| --- | --- | --- | --- | --- | --- | --- | --- | --- | --- |
| prediction | reference | | | | prediction | reference | | | |
|  | 0 days | 7 days | 14 days | 100 days |  | 0 days | 7 days | 14 days | 100 days |
| 0 days | **70** | 1 | 2 | 0 | 0 days | **124** | 0 | 3 | 0 |
| 7 days | 0 | **86** | 0 | 1 | 7 days | 13 | **119** | 13 | 0 |
| 14 days | 0 | 0 | **114** | 0 | 14 days | 3 | 21 | **132** | 5 |
| 100 days | 0 | 3 | 4 | **99** | 100 days | 0 | 0 | 2 | **205** |

Confusion table of Raman (“RS”) and FT-IR data for the PCA-LDA classification model for the cultivation times (in days) of contact inhibited quiescent cells.
