# Supplementary material for: Raman and infrared spectroscopy reveal that proliferating and quiescent human fibroblast cells age by biochemically similar but not identical processes
Source: PLoS One. 2018 Dec 3;13(12):e0207380. doi: 10.1371/journal.pone.0207380 (PMC6277109; doi:10.1371/journal.pone.0207380)
Supplement: S6 Table — Ten-fold cross-validation of PLS-LDA with 100 iterations for the kind of quiescent induction (contact inhibition or serum starvation) after 14 and 100 days without proliferating cells recovered from quiescence. Values for the Raman (“RS”) and FT-IR data are given in percentage. (DOCX) [file pone.0207380.s006.docx]

**S6 Table. Cross-validation of Raman and infrared spectra for the type of quiescent induction.**

|  | accuracy |  | contact inhibition | serum starvation |
| --- | --- | --- | --- | --- |
| RS | 99.3 | sensitivity | 100.0 | 97.7 |
|  |  | specificity | 97.7 | 100.0 |
| FT-IR | 96.6 | sensitivity | 98.0 | 95.4 |
|  |  | specificity | 95.4 | 98.0 |

Ten-fold cross-validation of PLS-LDA with 100 iterations for the kind of quiescent induction (contact inhibition or serum starvation) after 14 and 100 days without proliferating cells recovered from quiescence. Values for the Raman (“RS”) and FT-IR data are given in percentage.
