# Supplementary material for: Raman and infrared spectroscopy reveal that proliferating and quiescent human fibroblast cells age by biochemically similar but not identical processes
Source: PLoS One. 2018 Dec 3;13(12):e0207380. doi: 10.1371/journal.pone.0207380 (PMC6277109; doi:10.1371/journal.pone.0207380)
Supplement: S7 Table — Ten-fold cross-validation of PLS-LDA with 100 iterations of contact inhibited quiescent cells and the same cells recovered from G0 phase after 14 and 100 days. Values for the Raman (“RS”) and FT-IR data are given in percentage. (DOCX) [file pone.0207380.s007.docx]

**S7 Table. Cross-validation of Raman and infrared spectra of proliferating cells recovered from quiescence.**

|  | accuracy |  | quiescent cells | recovered cells from G_0_ phase |
| --- | --- | --- | --- | --- |
| RS | 95.5 | sensitivity | 97.0 | 93.3 |
|  |  | specificity | 93.3 | 97.0 |
| FT-IR | 93.7 | sensitivity | 91.3 | 96.3 |
|  |  | specificity | 96.3 | 91.3 |

Ten-fold cross-validation of PLS-LDA with 100 iterations of contact inhibited quiescent cells and the same cells recovered from G_0_ phase after 14 and 100 days. Values for the Raman (“RS”) and FT-IR data are given in percentage.
