# Supplementary material for: Raman and infrared spectroscopy reveal that proliferating and quiescent human fibroblast cells age by biochemically similar but not identical processes
Source: PLoS One. 2018 Dec 3;13(12):e0207380. doi: 10.1371/journal.pone.0207380 (PMC6277109; doi:10.1371/journal.pone.0207380)
Supplement: S8 Table — Ten-fold cross-validation of PLS-LDA with 100 iterations for the cell states (proliferation, senescence and 100 days contact inhibited quiescent cells) without proliferating cells recovered from quiescence. Values for the Raman (“RS”) and FT-IR data are given in percentage. (DOCX) [file pone.0207380.s008.docx]

**S8 Table. Cross-validation of Raman and infrared spectra of three cell states.**

|  | accuracy |  | proliferation | senescence | quiescence |
| --- | --- | --- | --- | --- | --- |
| RS | 99.5 | sensitivity | 99.0 | 100.0 | 99.4 |
|  |  | specificity | 99.6 | 100.0 | 99.5 |
| FT-IR | 99.7 | sensitivity | 100.0 | 99.3 | 100.0 |
|  |  | specificity | 99.7 | 100.0 | 100.0 |

Ten-fold cross-validation of PLS-LDA with 100 iterations for the cell states (proliferation, senescence and 100 days contact inhibited quiescent cells) without proliferating cells recovered from quiescence. Values for the Raman (“RS”) and FT-IR data are given in percentage.
