# Supplementary material for: Raman and infrared spectroscopy reveal that proliferating and quiescent human fibroblast cells age by biochemically similar but not identical processes
Source: PLoS One. 2018 Dec 3;13(12):e0207380. doi: 10.1371/journal.pone.0207380 (PMC6277109; doi:10.1371/journal.pone.0207380)
Supplement: S1 Fig — BJ cell states: (A) a proliferating cell (PD 28), (B) contact inhibited quiescent cells (100 days cultivation), (C) a serum starved quiescent cell (100 days cultivation) and (D) a senescent cell (PD 70). Images based on the C-H stretching region (2800 to 3020 cm-1) and the scale bars are (A) 5 μm and (B–D) 10 μm. (DOCX) [file pone.0207380.s009.docx]

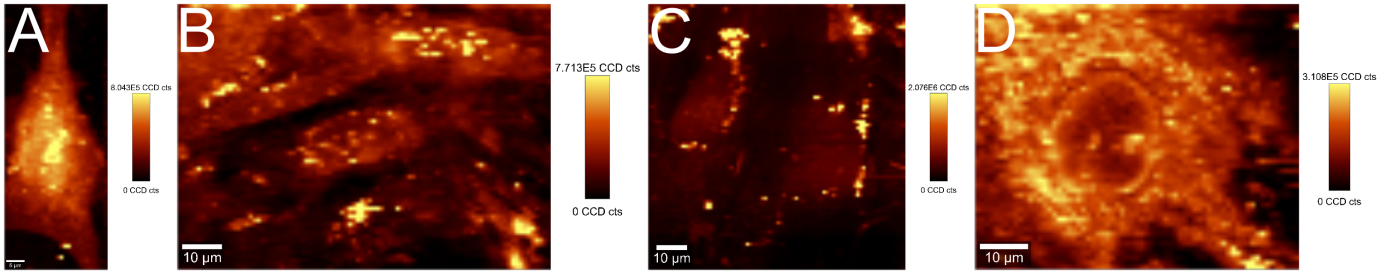


**S1 Fig. Raman images of three fibroblast cell states.**

BJ cell states: (A) a proliferating cell (PD 28), (B) contact inhibited quiescent cells (100 days cultivation), (C) a serum starved quiescent cell (100 days cultivation) and (D) a senescent cell (PD 70). Images based on the C-H stretching region (2800 to 3020 cm^‑1^) and the scale bars are (A) 5 µm and (B–D) 10 µm.
