# Supplementary material for: Raman and infrared spectroscopy reveal that proliferating and quiescent human fibroblast cells age by biochemically similar but not identical processes
Source: PLoS One. 2018 Dec 3;13(12):e0207380. doi: 10.1371/journal.pone.0207380 (PMC6277109; doi:10.1371/journal.pone.0207380)
Supplement: S2 Fig — Mean and standard deviation of (A) Raman and (B) FT-IR spectra of contact inhibited quiescent cells (BJ PD 28) for the cultivation times 0, 7, 14 and 100 days. The 0, 7, 14 and 100 days cultivated cells were displayed by different line styles. For a better visualization, the low wavenumber region from 600–1800 cm-1 in (A) is plotted 3fold enhanced. (DOCX) [file pone.0207380.s010.docx]

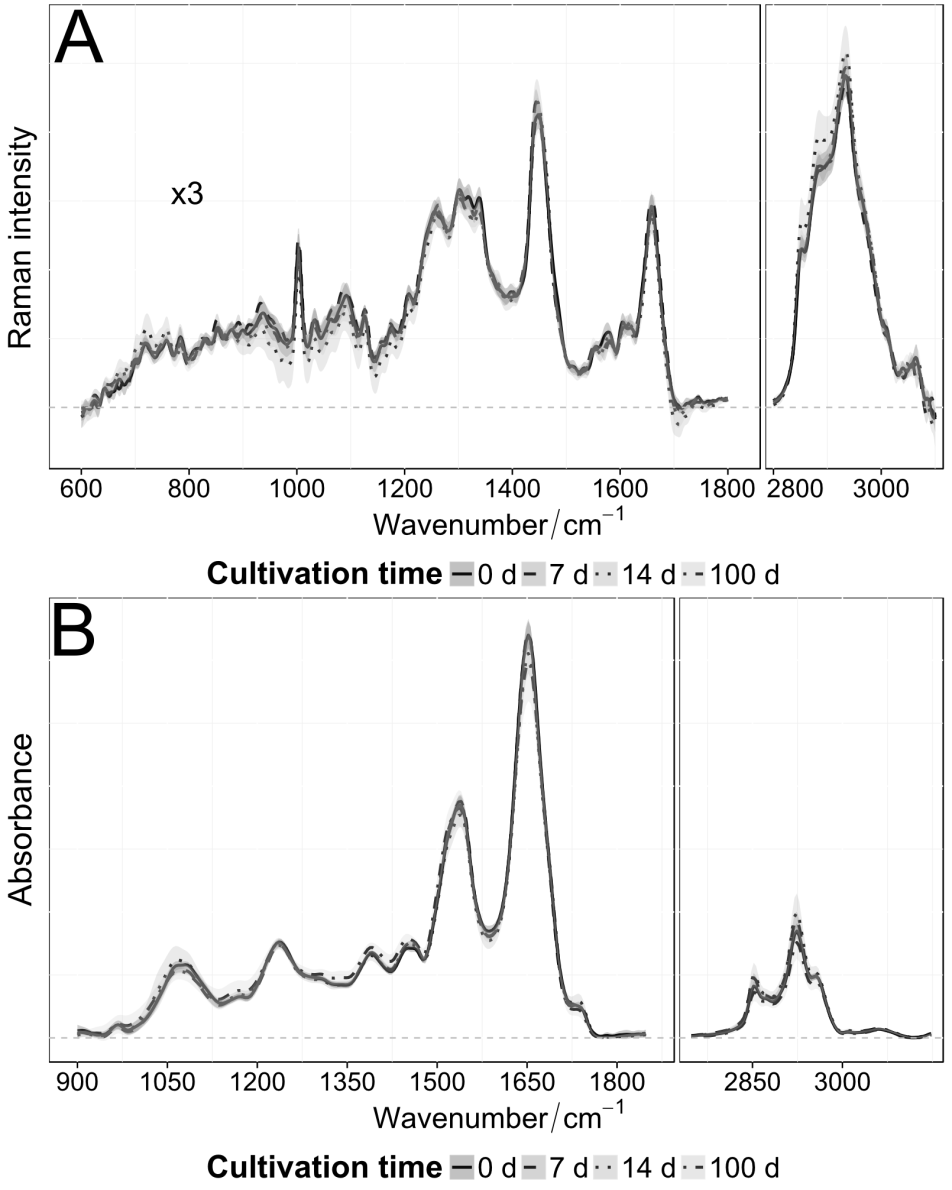


**S2 Fig. Raman and infrared spectra of quiescent cells with various cultivation times.**

Mean and standard deviation of (A) Raman and (B) FT-IR spectra of contact inhibited quiescent cells (BJ PD 28) for the cultivation times 0, 7, 14 and 100 days. The 0, 7, 14 and 100 days cultivated cells were displayed by different line styles. For a better visualization, the low wavenumber region from 600–1800 cm^‑1^ in (A) is plotted 3fold enhanced.
