# Supplementary material for: Raman and infrared spectroscopy reveal that proliferating and quiescent human fibroblast cells age by biochemically similar but not identical processes
Source: PLoS One. 2018 Dec 3;13(12):e0207380. doi: 10.1371/journal.pone.0207380 (PMC6277109; doi:10.1371/journal.pone.0207380)
Supplement: S3 Fig — Mean and standard deviation of (A) Raman and (B) FT-IR spectra of contact inhibited (dotted line) and serum starved (solid line) quiescent fibroblast cells (BJ PD 28) after 14 days (top) and 100 days (below) cultivation. For a better visualization, the low wavenumber region from 600–1800 cm-1 in (A) is plotted enhanced 3fold. (DOCX) [file pone.0207380.s011.docx]

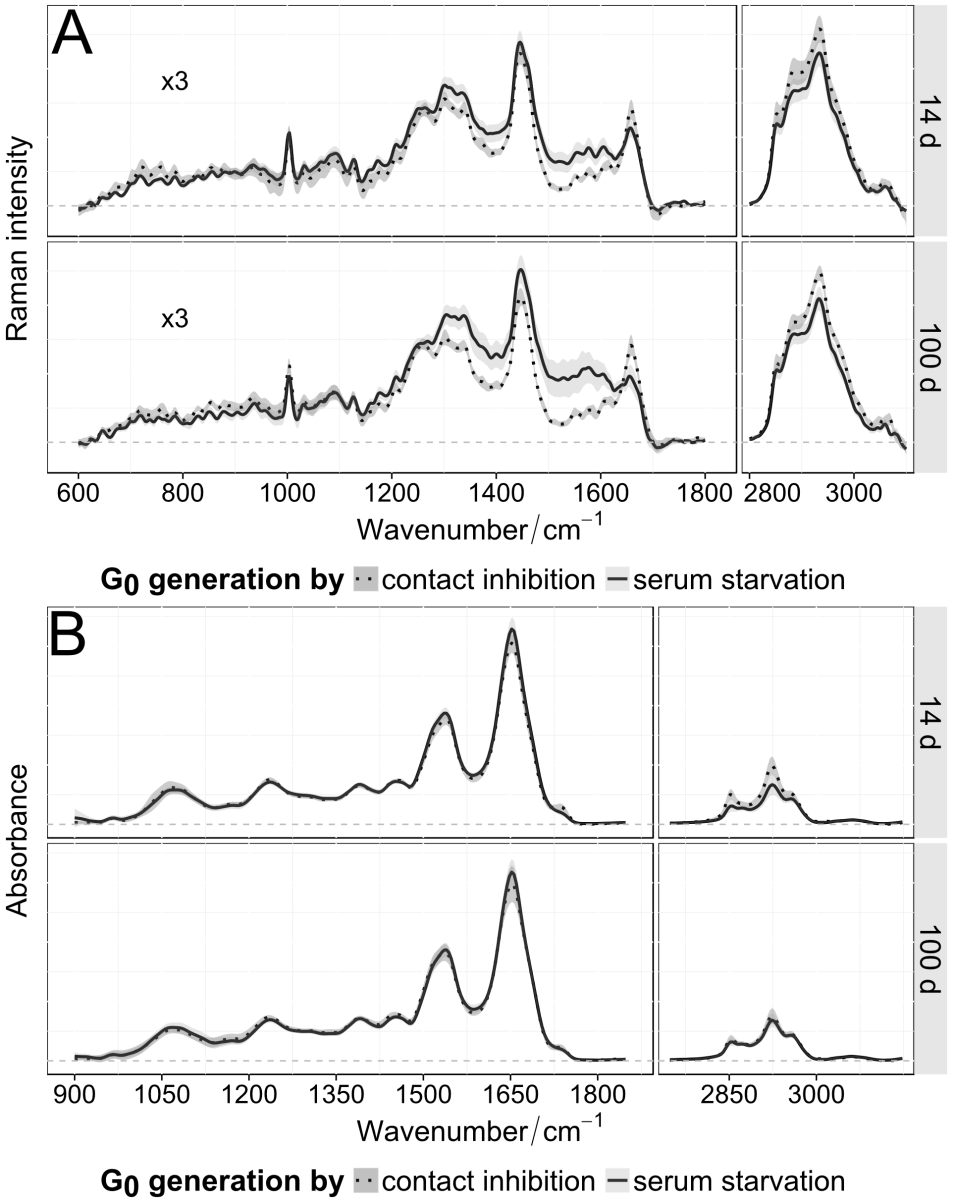


**S3 Fig. Raman and infrared spectra for the type of quiescent induction.**

Mean and standard deviation of (A) Raman and (B) FT-IR spectra of contact inhibited (dotted line) and serum starved (solid line) quiescent fibroblast cells (BJ PD 28) after 14 days (top) and 100 days (below) cultivation. For a better visualization, the low wavenumber region from 600–1800 cm^‑1^ in (A) is plotted enhanced 3fold.
