# Supplementary material for: Raman and infrared spectroscopy reveal that proliferating and quiescent human fibroblast cells age by biochemically similar but not identical processes
Source: PLoS One. 2018 Dec 3;13(12):e0207380. doi: 10.1371/journal.pone.0207380 (PMC6277109; doi:10.1371/journal.pone.0207380)
Supplement: S4 Fig — Mean and standard deviation of (A) Raman and (B) FT-IR spectra of contact inhibited quiescent cells (dotted line) and the same cells after recovery from quiescence (solid line) after 14 days (top) and 100 days (bottom) cultivation. The standard deviation is in gray (darker for quiescent cells and brighter for once again proliferating cells) and less pronounced. For a better visualization the low wavenumber region from 600–1800 cm-1 in (A) is plotted 3fold enhanced. (DOCX) [file pone.0207380.s012.docx]

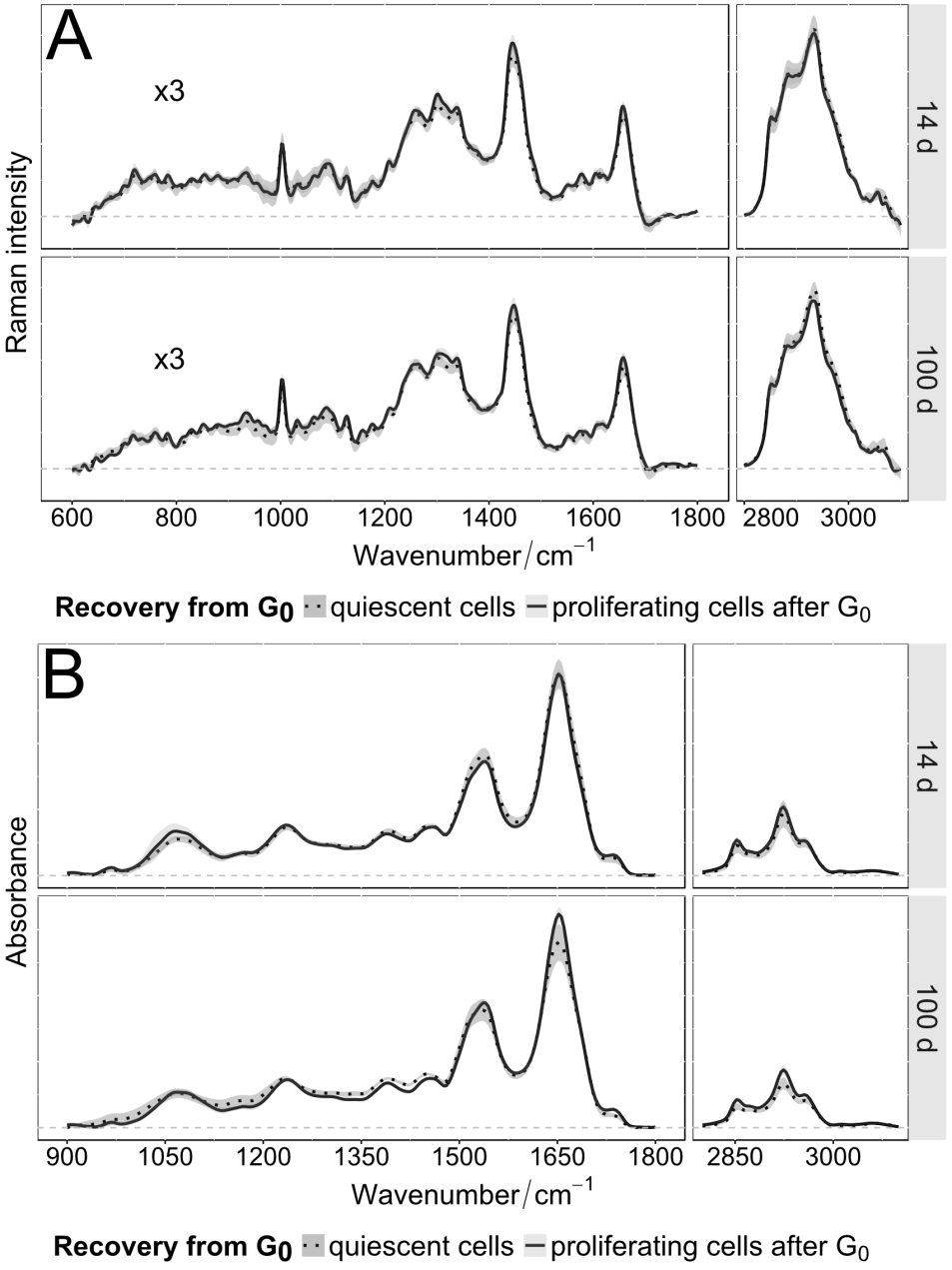


**S4 Fig. Raman and infrared spectra of proliferating cells recovered from quiescence versus quiescence.**

Mean and standard deviation of (A) Raman and (B) FT-IR spectra of contact inhibited quiescent cells (dotted line) and the same cells after recovery from quiescence (solid line) after 14 days (top) and 100 days (bottom) cultivation. The standard deviation is in gray (darker for quiescent cells and brighter for once again proliferating cells) and less pronounced. For a better visualization the low wavenumber region from 600–1800 cm^‑1^ in (A) is plotted 3fold enhanced.
