# Supplementary material for: Raman and infrared spectroscopy reveal that proliferating and quiescent human fibroblast cells age by biochemically similar but not identical processes
Source: PLoS One. 2018 Dec 3;13(12):e0207380. doi: 10.1371/journal.pone.0207380 (PMC6277109; doi:10.1371/journal.pone.0207380)
Supplement: S5 Fig — For quiescent cells (14 and 100 days contact inhibition) and proliferating cells recovered from quiescence (after 14 and 100 days contact inhibition, cells proliferating for 3 days), the 1658 cm−1 Raman band intensities (A, amide I proteins, C = C stretch) were plotted (with a fitted linear calibration, R2 = 0.17). Also, the 1338 cm-1 Raman band intensities (B, amide III proteins) were plotted (with a fitted linear calibration, R2 = 0.25). In total, 386 spectra were used for (A) and (B). Furthermore, in (C) FT-IR the absorption band at 1652 cm-1 (amide I, proteins) was related to 1446 cm-1 (proteins (asymmetric bending of methyl groups (CH3)) and/or lipids (CH2 scissoring of acyl chains)). In (D), FT-IR band ratios of 1652 cm-1 (amide I, proteins) versus 1540 cm-1 (amide II) are displayed. A linear calibration was fitted for (C, R2 = 0.41) and for (D, R2 = 0.53). In total, 694 spectra were used for (C) and (D). (DOCX) [file pone.0207380.s013.docx]

**
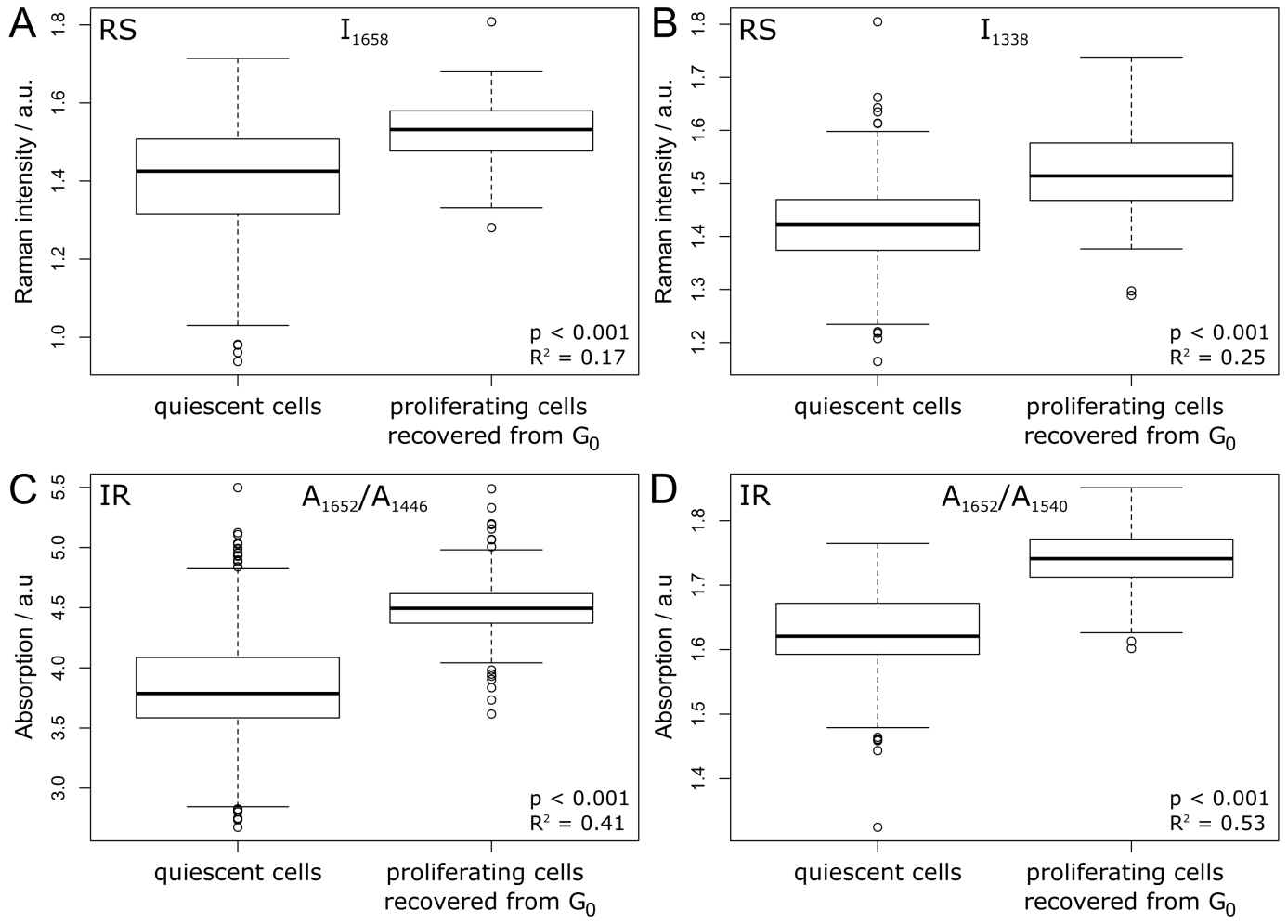
**

**S5 Fig. Raman and infrared spectroscopy ratio analyses of mostly proteins for quiescent cells and proliferating cells recovered from quiescence.**

For quiescent cells (14 and 100 days contact inhibition) and proliferating cells recovered from quiescence (after 14 and 100 days contact inhibition, cells proliferating for 3 days), the 1658 cm^−1^ Raman band intensities (A, amide I proteins, C=C stretch) were plotted (with a fitted linear calibration, R^2^ = 0.17). Also, the 1338 cm^‑1^ Raman band intensities (B, amide III proteins) were plotted (with a fitted linear calibration, R^2^ = 0.25). In total, 386 spectra were used for (A) and (B). Furthermore, in (C) FT-IR the absorption band at 1652 cm^-1^ (amide I, proteins) was related to 1446 cm^-1^ (proteins (asymmetric bending of methyl groups (CH_3_)) and/or lipids (CH_2_ scissoring of acyl chains)). In (D), FT-IR band ratios of 1652 cm^-1^ (amide I, proteins) versus 1540 cm^-1^ (amide II) are displayed. A linear calibration was fitted for (C, R^2^= 0.41) and for (D, R^2^ = 0.53). In total, 694 spectra were used for (C) and (D).
