# Supplementary material for: Raman and infrared spectroscopy reveal that proliferating and quiescent human fibroblast cells age by biochemically similar but not identical processes
Source: PLoS One. 2018 Dec 3;13(12):e0207380. doi: 10.1371/journal.pone.0207380 (PMC6277109; doi:10.1371/journal.pone.0207380)
Supplement: S6 Fig — Mean and standard deviation of (A) Raman and (B) FT-IR spectra of proliferating (BJ PD 28; dotted line), senescent (BJ PD 70; dashed line) and quiescent fibroblast cells (BJ PD 28 after 100 days contact inhibition; solid line). For a better visualization the low wavenumber region from 600–1800 cm-1 in (A) is plotted enhanced three-fold. (DOCX) [file pone.0207380.s014.docx]

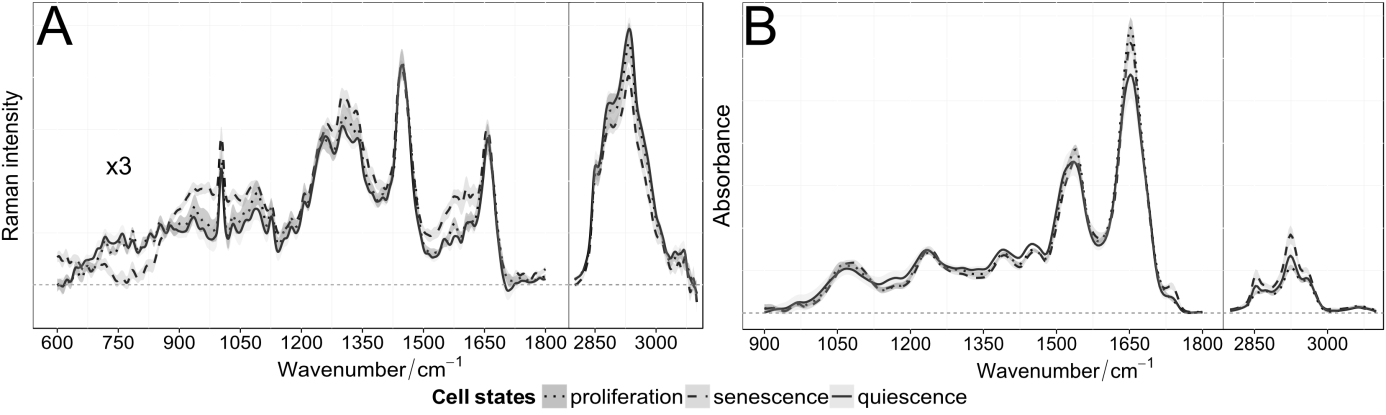


**S6 Fig. Raman and infrared spectra of three cell states.**

Mean and standard deviation of (A) Raman and (B) FT-IR spectra of proliferating (BJ PD 28; dotted line), senescent (BJ PD 70; dashed line) and quiescent fibroblast cells (BJ PD 28 after 100 days contact inhibition; solid line). For a better visualization the low wavenumber region from 600–1800 cm^‑1^ in (A) is plotted enhanced three-fold.
