# Supplementary material for: Raman and infrared spectroscopy reveal that proliferating and quiescent human fibroblast cells age by biochemically similar but not identical processes
Source: PLoS One. 2018 Dec 3;13(12):e0207380. doi: 10.1371/journal.pone.0207380 (PMC6277109; doi:10.1371/journal.pone.0207380)
Supplement: S7 Fig — The Raman intensity ratio (A) of the 1652 cm−1 band (amide I proteins, C = C stretch) to the 1454 cm−1 band (lipids, CH2 twist) is plotted with a fitted linear calibration (R2 = 0.33). Also for the DNA/RNA intensity (B) the comparison of the three cell states is plotted using the Raman band 782 cm-1 (cytosine, thymine & uracil ring breathing) with a fitted linear calibration (R2 = 0.66). In total, 253 spectra were used for (A) and (B). Furthermore, in (C) FT-IR the absorption band at 1652 cm-1 (amide I, proteins) was related to 1436 cm-1 (proteins (asymmetric bending of methyl groups (CH3)) and/or lipids (CH2 scissoring of acyl chains)). In (D), FT-IR band ratios of 1740 cm-1 (C = O stretching of ester functional groups, lipids) versus 1716 cm-1 (C = O stretching of base pairing in nucleic acids, RNA/DNA contents) were displayed. A linear calibration is fitted for (C, R2 = 0.27) and (D, R2 = 0.65). In total, 627 spectra were used for (C) and (D). (DOCX) [file pone.0207380.s015.docx]

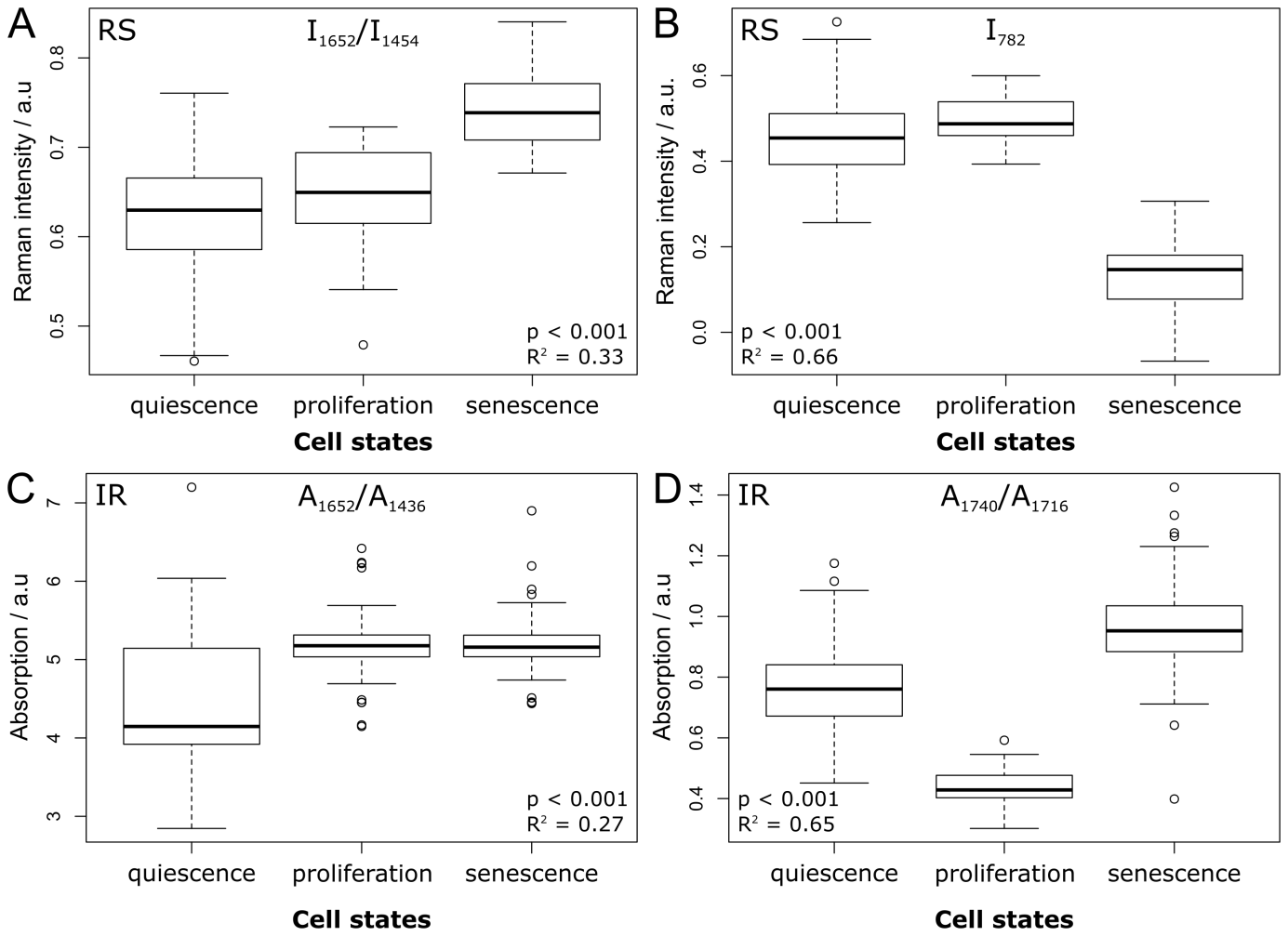


**S7 Fig. Raman and infrared spectroscopy ratio analyses for proteins, lipids and nucleic acids for the three cell states quiescence (100 days contact inhibited quiescent cells without recovery), proliferation and senescence.**

The Raman intensity ratio (A) of the 1652 cm^−1^ band (amide I proteins, C=C stretch) to the 1454 cm^−1^ band (lipids, CH_2_ twist) is plotted with a fitted linear calibration (R^2^ = 0.33). Also for the DNA/RNA intensity (B) the comparison of the three cell states is plotted using the Raman band 782 cm^-1^ (cytosine, thymine & uracil ring breathing) with a fitted linear calibration (R^2^ = 0.66). In total, 253 spectra were used for (A) and (B). Furthermore, in (C) FT‑IR the absorption band at 1652 cm^-1^ (amide I, proteins) was related to 1436 cm^-1^ (proteins (asymmetric bending of methyl groups (CH_3_)) and/or lipids (CH_2_ scissoring of acyl chains)). In (D), FT-IR band ratios of 1740 cm^-1^ (C=O stretching of ester functional groups, lipids) versus 1716 cm^‑1^ (C=O stretching of base pairing in nucleic acids, RNA/DNA contents) were displayed. A linear calibration is fitted for (C, R^2^ = 0.27) and (D, R^2^ = 0.65). In total, 627 spectra were used for (C) and (D).
