# Supplementary material for: Raman and infrared spectroscopy reveal that proliferating and quiescent human fibroblast cells age by biochemically similar but not identical processes
Source: PLoS One. 2018 Dec 3;13(12):e0207380. doi: 10.1371/journal.pone.0207380 (PMC6277109; doi:10.1371/journal.pone.0207380)
Supplement: S8 Fig — PLS-LDA scatter plot of Raman spectra (A) of proliferating cells (“0 d R0”) and 100 days contact-inhibited quiescent cells without recovery (“100 d R0”) and recovered from quiescence (“100 d R1”) compared to replicative-grown senescent cells (“220 d R0”). Cultivation time (0, 100 and 220 days) and proliferating cells having recovered from quiescence (“R1”, pale blue) were specified by color (0 days: orange, 100 days: blue, 220 days: red). Mean and difference spectra (“diff”, 0 d proliferation vs. 100 d quiescence “R1”) from the classified cells are plotted together with the PLS-LD1 and -LD2 coefficients vs. the wavenumber (B). (DOCX) [file pone.0207380.s016.docx]

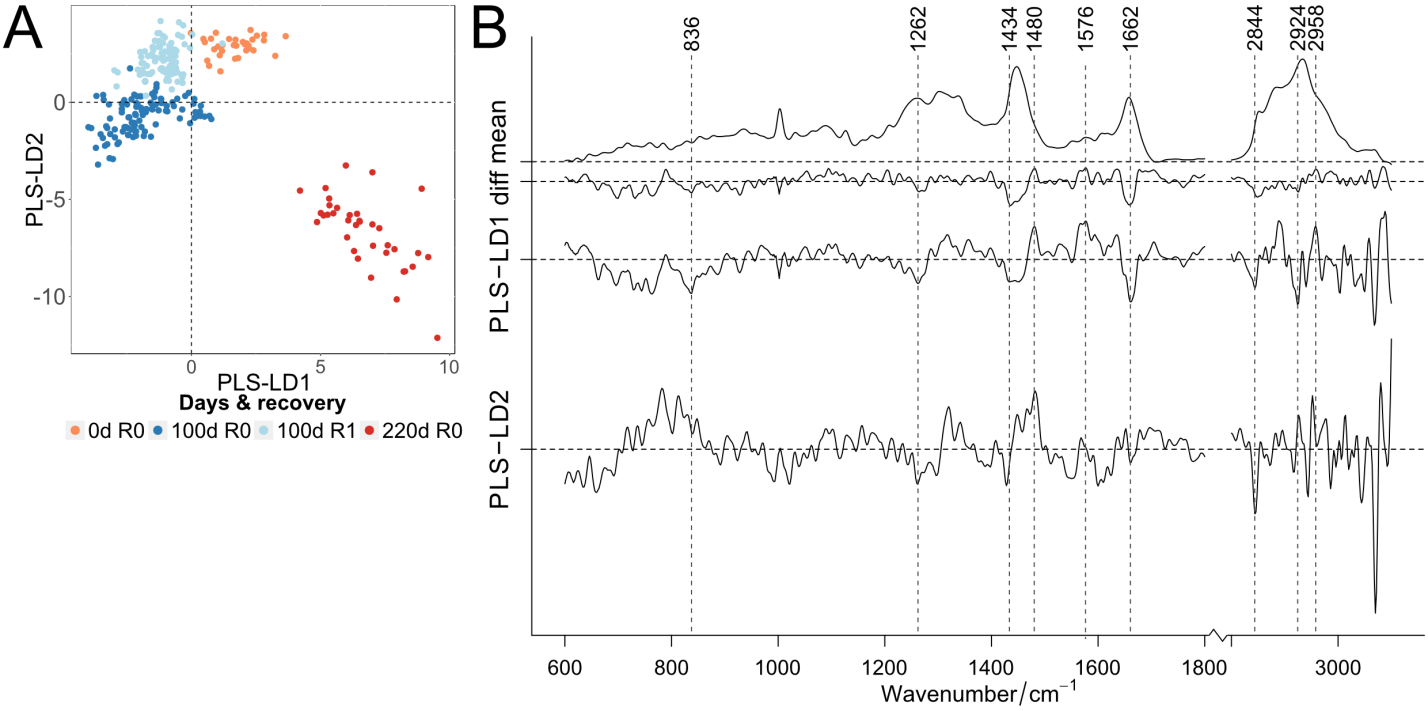


**S8 Fig. Classification of Raman spectra of proliferating, long-term cultivated quiescent and senescent cells.**

PLS-LDA scatter plot of Raman spectra (A) of proliferating cells (“0 d R0”) and 100 days contact-inhibited quiescent cells without recovery (“100 d R0”) and recovered from quiescence (“100 d R1”) compared to replicative-grown senescent cells (“220 d R0”). Cultivation time (0, 100 and 220 days) and proliferating cells having recovered from quiescence (“R1”, pale blue) were specified by color (0 days: orange, 100 days: blue, 220 days: red). Mean and difference spectra (“diff”, 0 d proliferation vs. 100 d quiescence “R1”) from the classified cells are plotted together with the PLS-LD1 and -LD2 coefficients vs. the wavenumber (B).
